# Fish Predation by Semi-Aquatic Spiders: A Global Pattern

**DOI:** 10.1371/journal.pone.0099459

**Published:** 2014-06-18

**Authors:** Martin Nyffeler, Bradley J. Pusey

**Affiliations:** 1 Section of Conservation Biology, Department of Environmental Sciences, University of Basel, Basel, Switzerland; 2 Centre for Excellence in Natural Resource Management, The University of Western Australia, Albany, Australia; Aarhus University, Denmark

## Abstract

More than 80 incidences of fish predation by semi-aquatic spiders – observed at the fringes of shallow freshwater streams, rivers, lakes, ponds, swamps, and fens – are reviewed. We provide evidence that fish predation by semi-aquatic spiders is geographically widespread, occurring on all continents except Antarctica. Fish predation by spiders appears to be more common in warmer areas between 40° S and 40° N. The fish captured by spiders, usually ranging from 2–6 cm in length, are among the most common fish taxa occurring in their respective geographic area (e.g., mosquitofish [*Gambusia* spp.] in the southeastern USA, fish of the order Characiformes in the Neotropics, killifish [*Aphyosemion* spp.] in Central and West Africa, as well as Australian native fish of the genera *Galaxias*, *Melanotaenia*, and *Pseudomugil*). Naturally occurring fish predation has been witnessed in more than a dozen spider species from the superfamily Lycosoidea (families Pisauridae, Trechaleidae, and Lycosidae), in two species of the superfamily Ctenoidea (family Ctenidae), and in one species of the superfamily Corinnoidea (family Liocranidae). The majority of reports on fish predation by spiders referred to pisaurid spiders of the genera *Dolomedes* and *Nilus* (>75% of observed incidences). There is laboratory evidence that spiders from several more families (e.g., the water spider *Argyroneta aquatica* [Cybaeidae], the intertidal spider *Desis marina* [Desidae], and the ‘swimming’ huntsman spider *Heteropoda natans* [Sparassidae]) predate fish as well. Our finding of such a large diversity of spider families being engaged in fish predation is novel. Semi-aquatic spiders captured fish whose body length exceeded the spiders’ body length (the captured fish being, on average, 2.2 times as long as the spiders). Evidence suggests that fish prey might be an occasional prey item of substantial nutritional importance.

## Introduction

A diverse array of predators feed on fish, including piscivorous fish, birds (e.g., egrets, herons, cormorants, gulls, osprey, kites, eagles), fish-eating bats, otters, bears, snakes, certain turtles, etc. [1]–[5]. Predation by a few large arthropods, that spend all, or at least part, of their life cycle in the aquatic environment and are generally well-adapted at catching aquatic prey such as small fish, tadpoles, frogs, etc., has also been documented [6]–[7]. For example, water scorpions (Nepidae), giant water-bugs (Belostomatidae), backswimmers (Notonectidae), and water boatmen (Corixidae) are known to kill and eat small fish [7]. A caddisfly species, *Plectrocnemia conspersa* (Polycentropodidae), has been observed preying on fish fry [6], and nymphs of the dragonfly *Cordulegaster dorsalis* (Cordulegastridae) have been reported to kill fish of >2.5 cm in length [7]. Furthermore, diving beetles (Dytiscidae) and scavenger water beetles (Hydrophilidae) often predate small fish [7], highlighting the plethora of predatory arthropods with trophic interactions with freshwater fish.

Another group of predaceous arthropods known to catch and eat small fish is spiders, particularly large, semi-aquatic pisaurid spiders of the genera *Dolomedes* and *Nilus* (‘fishing spiders’). The notion of fish-catching spiders is rather peculiar if we consider that spiders, as a whole, are traditionally viewed as the classic example of a predator that feeds on insects, yet some spiders are well-adapted for life near, or on, the water surface [8]. Despite the widespread assumption of spiders primarily being insectivores, piscivory is not altogether surprising considering that a number of spiders (e.g., Araneidae, Nephilidae, Pisauridae, Sparassidae, Theraphosidae, and Theridiidae) occasionally supplement their arthropod diet with small vertebrates including frogs, toads, salamanders, lizards, snakes, mice, rats, bats, and birds [7], [9]–[10] and many spiders may be found at the land-water interface. Photographic evidence supporting the existence of fish-catching by ‘fishing spiders’ has been published [11]–[13] but published accounts of open-field assessment of fish predation are often anecdotal, from very old literature sources and originate from only few locations [14]–[19]. Additionally, the majority of published photographic sources depict spiders preying upon fish in captivity [11]–[13]. More recently, evidence of the extent of fish predation by spiders in laboratory [20]–[24] and field experiments [25], suggests it is more widespread than traditionally thought. However, the propensity for spiders to feed on fish and the importance of this trophic relationship under natural conditions remains unclear. We conducted a global analysis of all available literature on fish predation by spiders and unpublished information from biologists and naturalists (arachnologists, ichthyologists, aquatic ecologists, photographers, etc.) to provide a broad, conceptual framework for this trophic relationship placed within the context of spider behavior and nutritional ecology.

## Methods

An extensive bibliographic search was conducted to locate information concerning fish predation by spiders. The search was based largely on the Thomson-Reuters database (Web of Science), Google Scholar, Google Books, and ProQuest Dissertations & Theses. In addition, an internet search for information on this topic was conducted; authors of photographic material and reports on fish predation by spiders were contacted to obtain detailed information on these observations. Furthermore, inquiries among biologists were undertaken for unpublished reports on this topic. A total of 89 incidences of fish predation by spiders was gathered (Table 1). For the most part, only incidences of fish predation by free living spiders are listed in Table 1; however, a few incidences where semi-aquatic spiders killed fish in aquaria after wandering into buildings (not-staged situations; [15], [18]) are included. Staged observations of captive spiders predating fish in aquaria, fish tanks, or garden pools are included in Tables 2 and 3. Fourty-four (49%) of the incidences in Table 1 were previously reported in the scientific literature and 44% of observations included photographic documentation of predation.

**Table 1:** Reports of fish predation by spiders under natural conditions in the field, based upon published literature and unpublished data (File S1 provides detailed documentation of all predation events).

Predator (spider taxon)		Prey (fish taxon)		Estimated total length of fish (cm)	Estimated fish length/spider length ratio	Type of evidence	Country	Source	report #
Species	Family	Species	Family						
*Agroeca lusatica*	Liocranidae	‘Trout’ (fry)	Salmonidae	2	2.8	Direct observation	France	[165]	86
*Ancylometes bogotensis*	Ctenidae	Unidentified	Poeciliidae	N/A	N/A	Photo	Costa Rica	[166]	47
*Ancylometes rufus*	Ctenidae	Unidentified	Unidentified	N/A	N/A	Direct observation	Brazil	[35]	58
*Ancylometes* sp.	Ctenidae	*Cyphocharax* sp.	Curimatidae (Characiformes)	6	2.4	Photo	Ecuador	Ed Germain, pers. comm.	52
*Ancylometes* sp.	Ctenidae	Unidentified	Characiformes	N/A	N/A	Photo	Ecuador	Tim Wohlberg, pers. comm. http://zufalladventures.com/	53
*Ancylometes* sp.	Ctenidae	Unidentified	Characiformes	N/A	N/A	Photo	Ecuador	Cleatus Cobb, flickr website	54
*Ancylometes* sp.	Ctenidae	Unidentified	Characiformes	N/A	N/A	Photo	Ecuador	http://alexlisasouthamerica.blogspot.ch	55
*Ancylometes* sp.?	Ctenidae	Unidentified	Unidentified	N/A	N/A	Direction observation	Ecuador	http://www.tripadvisor.de	56
*Ancylometes* sp.	Ctenidae	Unidentified	Characiformes	∼7–8	2.7	Photo	Peru	http://thinkjungle.com/amazon-rainforest-life/amazon-rainforest-carnivores/	60
*Dolomedes facetus*	Pisauridae	*Carassius auratus*	Cyprinidae	9	3.7	Photo	Australia (Sydney)	[19]	64
*Dolomedes facetus*	Pisauridae	*Xiphophorus* sp.	Poeciliidae	5.5	2.2	Photo	Australia (Brisbane area)	Peter Liley, pers. comm.	67
*Dolomedes facetus*	Pisauridae	Unidentified	Unidentified	N/A	N/A	Direct observation	Australia (Audley, NSW)	[19]	63
*Dolomedes facetus*	Pisauridae	Unidentified	Unidentified	N/A	N/A	Photo	Australia	Jean-Paul Ferrero, ardeaprints.com	70
*Dolomedes mizhoanus*	Pisauridae	Unidentified	Unidentified	N/A	N/A	Direct observation	China	[105]	77
*Dolomedes okefinokensis*	Pisauridae	*Gambusia holbrooki*	Poeciliidae	4	1.8	Photo	USA (Florida, Big Cypress National Preserve)	Misti Little, pers. comm.	28
*Dolomedes okefinokensis*	Pisauridae	*Gambusia affinis*	Poeciliidae	2.5	1.0	Direct observation	USA (Florida, near Lake Washington)/Incidence 1	[121] (ID of spider changed by Carico [29])	21
*Dolomedes okefinokensis*	Pisauridae	*Gambusia affinis*	Poeciliidae	2.5	1.0	Direct observation	USA (Florida/near Lake Washington)/Incidence 2	[121] (ID of spider changed by Carico [29])	22
*Dolomedes okefinokensis*	Pisauridae	*Gambusia affinis*	Poeciliidae	2.5	1.0	Direct observation	USA (Florida/near Lake Washington)/Incidence 3	[121] (ID of spider changed by Carico [29])	23
*Dolomedes plantarius*	Pisauridae	*Gambusia holbrooki*	Poeciliidae	N/A	N/A	Direct observation	Italy	Emanuele Biggi, pers. comm.	85
*Dolomedes plantarius*	Pisauridae	*Pungitius laevis*	Gasterosteidae	N/A	N/A	Photo	UK/Incidence 1	Helen Smith, pers. comm.	87
*Dolomedes plantarius*	Pisauridae	*Pungitius laevis*	Gasterosteidae	N/A	N/A	Direct observation	UK/Incidence 2	Helen Smith, pers. comm.	88
*Dolomedes raptor*	Pisauridae	Unidentified	Infraclass Teleostei	5.5	2.2	Photo	Taiwan	I-Min To, pers. comm.	74
*Dolomedes saganus*	Pisauridae	*Oryzias curvinotus*	Adrianichthyidae	4.3	1.8	Direct observation	Hong Kong	[167]	75
*Dolomedes saganus*	Pisauridae	*Pseudorasbora parva*	Cyprinidae	3.3	1.4	Direct observation	Japan	[168]	73
*Dolomedes scriptus*	Pisauridae	‘Darter’	Percidae	N/A	N/A	Direct observation	USA (unspecified location)	[29]	44
*Dolomedes scriptus*	Pisauridae	*Lepomis cyanellus*	Centrarchidae	5.5	2.5	Photo	Canada (Ontario)	Lloyd Alter, pers. comm.	46
*Dolomedes scriptus*	Pisauridae	Unidentified	Infraclass Teleostei	N/A	N/A	Photo	USA (unspecified location)	http://www.flickr.com/photos/44608110@N06/4114197379/	45
*Dolomedes scriptus*	Pisauridae	‘Minnow’	Cyprinidae	N/A	N/A	Photo	USA (Michigan)	[169]	41
*Dolomedes tenebrosus?*	Pisauridae	*Notemigonus crysoleucas*	Cyprinidae	N/A	N/A	Photo	USA (Maine)	Jeffrey Hollis, pers. comm.	40
*Dolomedes tenebrosus*	Pisauridae	*Semotilus atromaculatus*	Cyprinidae	6.5	3.0	Photo	USA (Kentucky)	Jason Butler, pers. comm.	33
*Dolomedes triton*	Pisauridae	*Carassius auratus*	Cyprinidae	5	2.2	Photo	USA (Texas)	Leslie Todd, flickr website	11
*Dolomedes triton*	Pisauridae	*Gambusia holbrooki*	Poeciliidae	4.5	2.0	Photo	USA (Florida, Lady Lake)	Machele White, pers. comm.	20
*Dolomedes triton*	Pisauridae	*Gambusia* sp.	Poeciliidae	N/A	N/A	Photo	USA (Florida, near Tampa)	Stacy Cyrus, DavesGarden website	24
*Dolomedes triton?*	Pisauridae	*Gambusia affinis*	Poeciliidae	N/A	N/A	Photo	USA (Florida, Washington County)	Paul Moler, pers. comm.	17
*Dolomedes triton*	Pisauridae	*Gambusia affinis*	Poeciliidae	N/A	N/A	Unknown	USA (Mississippi)	[170]	15
*Dolomedes triton*	Pisauridae	*Gambusia holbrooki*	Poeciliidae	5	1.8	Photo	USA (North Carolina)	Patrick Randall, pers. comm.	30
*Dolomedes triton*	Pisauridae	*Gila ditaenia*	Cyprinidae	N/A	N/A	Photo	USA (Arizona)	Andreas Kettenburg, pers. comm.	9
*Dolomedes triton*	Pisauridae	*Fundulus chrysotus*	Fundulidae	3	1.2	Photo	USA (Texas)	Richard Dashnau, pers. comm.	10
*Dolomedes triton*	Pisauridae	*Heterandria formosa*	Poeciliidae	N/A	N/A	Photo	USA (Florida, Tsala Apopka Lake)	Claire Sunquist-Blunden, pers. comm.	19
*Dolomedes triton*	Pisauridae	*Ictalurus punctatus* (fingerling)	Ictaluridae	≤6	≤3	Direct observation	USA (Oklahoma)	[131]	12
*Dolomedes triton*	Pisauridae	Unidentified	Unidentified	N/A	N/A	Direct observation	USA (Florida, Highlands Hammock State Park)	Brian Kenney, pers. comm.	25
*Dolomedes triton*	Pisauridae	Unidentified	Unidentified	N/A	N/A	Direct observation	USA (Florida, near Bradenton)	Brian Kenney, pers. comm.	26
*Dolomedes triton*	Pisauridae	Unidentified	Unidentified	N/A	N/A	Direct observation	USA (Florida, near Venice)	Brian Kenney, pers. comm.	27
*Dolomedes triton*	Pisauridae	Unidentified	Unidentified	2.5	1.3	Direct observation	USA (New York)	[171] (ID by Carico [29])	39
*Dolomedes vittatus*	Pisauridae	Unidentified	Fundulidae?	5.5	2.5	Photo	USA (unspecified location)	[172]	43
*Dolomedes* sp.	Pisauridae	*Carassius auratus*	Cyprinidae	7	3.0	Direct observation	Australia (Adelaide)/Incidence 1	[19]	61
*Dolomedes* sp.	Pisauridae	*Carassius auratus*	Cyprinidae	7.5	3.0	Photo	Australia (Adelaide)/Incidence 2	[19]	62
*Dolomedes* sp.	Pisauridae	*Carassius auratus*	Cyprinidae	2.5	1.0	Direct observation	Australia (Lismore)	[19]	65
*Dolomedes* sp.	Pisauridae	*Elassoma zonatum*	Elassomatidae	3.5	1.8	Direct observation	USA (California)/Incidence 1	[18], [173]	1
*Dolomedes* sp.	Pisauridae	*Elassoma zonatum*	Elassomatidae	3.5	1.8	Direct observation	USA (California)/Incidence 2	[18], [173]	2
*Dolomedes* sp.	Pisauridae	*Elassoma zonatum*	Elassomatidae	3.5	1.8	Direct observation	USA (California)/Incidence 3	[18], [173]	3
*Dolomedes* sp.	Pisauridae	*Elassoma zonatum*	Elassomatidae	3.5	1.8	Direct observation	USA (California)/Incidence 4	[18], [173]	4
*Dolomedes* sp.	Pisauridae	*Elassoma zonatum*	Elassomatidae	3.5	1.8	Direct observation	USA (California)/Incidence 5	[18], [173]	5
*Dolomedes* sp.	Pisauridae	*Elassoma zonatum*	Elassomatidae	3.5	1.8	Direct observation	USA (California)/Incidence 6	[18], [173]	6
*Dolomedes* sp.	Pisauridae	*Elassoma zonatum*	Elassomatidae	3.5	1.8	Direct observation	USA (California)/Incidence 7	[18], [173]	7
*Dolomedes* sp.	Pisauridae	*Elassoma zonatum*	Elassomatidae	3.5	1.8	Direct observation	USA (California)/Incidence 8	[18], [173]	8
*Dolomedes* sp. (not *facetus*)	Pisauridae	*Galaxias olidus*	Galaxiidae	7.5	3.4	Photo	Australia (Goomburra)	Loren Jarvis, pers. comm.	66
*Dolomedes* sp.	Pisauridae	*Gambusia affinis*	Poeciliidae	N/A	N/A	Direct observation	USA (Kentucky)	[156]	35
*Dolomedes* sp.	Pisauridae	*Gambusia affinis*	Poeciliidae	N/A	N/A	Direct observation	USA (Kentucky)	Jason Butler, pers. comm.	34
*Dolomedes* sp.	Pisauridae	*Gambusia affinis*	Poeciliidae	N/A	N/A	Direct observation	USA (Louisiana)	[131]	13
*Dolomedes* sp.	Pisauridae	*Lepomis macrochirus*	Centrarchidae	N/A	N/A	Direct observation	USA (Louisiana)	[131]	14
*Dolomedes* sp.	Pisauridae	‘Minnow’	Cyprinidae	7.6	3.8	Direct observation	USA (Alabama)	[15]	16
*Dolomedes* sp.	Pisauridae	‘Minnow’	Cyprinidae	3.2	1.6	Photo	USA (Georgia)	[18]	29
*Dolomedes* sp.	Pisauridae	‘Minnow’	Cyprinidae	8.3	4.1	Direct observation	USA (New Jersey)	[15]	36
*Dolomedes* sp.	Pisauridae	*Oncorhynchus mykiss* (fry)	Salmonidae	N/A	N/A	Direct observation	USA (Tennessee)	[17]	32
*Dolomedes* sp.	Pisauridae	*Oncorhynchus mykiss* (fingerling)	Salmonidae	5.7	3.0	Direct observation	USA (Tennessee)	[17]	31
*Dolomedes* sp.	Pisauridae	Unidentified	Centrarchidae	5	2.5	Direct observation	USA (Pennsylvania)/Incidence 1	[15]	37
*Dolomedes* sp.	Pisauridae	Unidentified	Centrarchidae	5	2.5	Direct observation	USA (Pennsylvania)/Incidence 2	[15]	38
*Dolomedes* sp.	Pisauridae	Unidentified	Unidentified	N/A	N/A	Direct observation	USA (northern Florida)	Paul Moler, pers. comm.	18
*Dolomedes* sp.	Pisauridae	*Micropterus dolomieu*	Centrarchidae	4.5	2.0	Photo	USA (Wisconsin)	Tod Lewis, pers. comm.	42
*Nilus curtus*	Pisauridae	Unidentified	Unidentified	probably >4	2.0	Direct observation	South Africa	Astri Leroy, pers. comm.	79
*Nilus massajae*	Pisauridae	Unidentified	Unidentified	4	1.7	Photo	Zimbabwe	Marcelo de Freitas, pers. comm.	81
*Nilus* sp.	Pisauridae	*Aphyosemion* sp.	Nothobranchiidae	4.5	2.2	Photo	Cameroon	Duncan Reid, pers. comm.	82
*Nilus* sp.	Pisauridae	*Aphyosemion walker*	Nothobranchiidae	3	1.5	Photo	Ivory Coast/Incidence 1	[174]	83
*Nilus* sp.	Pisauridae	*Aphyosemion walker*	Nothobranchiidae	N/A	N/A	Direct observation	Ivory Coast/other incidences	[174]	84
*Nilus* sp.	Pisauridae	‘Trout’ (fry)	Salmonidae	N/A	N/A	Direct observation	South Africa	[14]	80
*Pardosa pseudoannulata*	Lycosidae	*Elassoma zonatum*	Elassomatidae	1.9	1.9	Direct observation	India	[82]	78
*Trechalea sp.*	Trechaleidae	Unidentified	Characiformes	N/A	1.8	Photo	Brazil	Jacques Jangoux, pers. comm.	59
*Trechalea sp.*	Trechaleidae	Unidentified	Characiformes	N/A	1.9	Photo	Colombia	Juan Esteban Arias A., pers. comm.	50
*Trechalea* sp.	Trechaleidae	Unidentified	Characiformes	N/A	2.2	Photo	Colombia	Solimary Garcia Hernandez, pers. comm.	51
*Trechalea* sp.	Trechaleidae	Unidentified	Characiformes	N/A	2.2	Photo	Panama	Jessica Stapley, pers. comm.	48
Unidentified	Ctenidae	Unidentified	Siluriformes (most likely Pimelodidae)	N/A	N/A	Photo	Ecuador	Craig Harrison, travel blog/ecuador	57
Unidentified	Trechaleidae?	Unidentified	Characiformes	N/A	N/A	Direct observation	Panama	[17]	49
Unidentified	Pisauridae	*Cyprinus carpio*	Cyprinidae	2	1.0	Direct observation	Australia (Victoria)	Alison King, pers. comm.	89
Unidentified	Pisauridae	*Melanotaenia* spp.	Melanotaeniidae	N/A	N/A	Direct observation	Australia (Cairns)	Bradley Pusey, pers. comm.	68
Unidentified	Pisauridae	*Parazacco spilurus*	Cyprinidae	2.5	N/A	Direct observation	Hong Kong	David Dudgeon, pers. comm.	76
Unidentified	Pisauridae	*Pseudomugil* spp.	Pseudomugilidae	N/A	N/A	Direct observation	Australia (Cairns)	Bradley Pusey, pers. comm.	69
Unidentified	Pisauridae	*Rasbora calliura*	Cyprinidae	6	3.5	Photo	Borneo	Michael Lo, pers. comm.	72
Unidentified	Pisauridae?	Unidentified	Unidentified	N/A	N/A	Direct observation	Australia	Morsten 1835 (cited by [19])	71

**Table 2:** Fresh weight and body length (cephalothorax plus abdomen) of adult spider species reported to catch fish.

Spider species	Spider family	Weight (g)	Body length (cm)	Location of observation	Source	report #
*Agroeca lusatica*	Liocranidae	N/A	∼0.7	Wild	[30], [165]	86
*Ancylometes bogotensis*	Ctenidae	1.4–2.6	2	Wild	[22], [35]	47
*Ancylometes rufus*	Ctenidae	≤7	3–4	Wild	[35], [41]	58
*Argyroneta aquatica*	Cybaeidae	∼0.1–0.3	1.2–1.9	Captivity	[23], [38], [88]	
*Desis marina*	Desidae	∼0.1	1–1.5	Captivity	[19], [31]–[32]	
*Dolomedes dondalei*	Pisauridae	N/A	2.5	Captivity	[11]	
*Dolomedes facetus*	Pisauridae	N/A	2	Wild	[19]	63–64, 67, 70
*Dolomedes fimbriatus*	Pisauridae	∼0.5	2	Captivity	[13], [23], [89], [175]	
*Dolomedes mizhoanus*	Pisauridae	N/A	2.4	Wild	[40]	77
*Dolomedes okefinokensis*	Pisauridae	∼2*	2.5	Wild	[27], [29]	21–23, 28
*Dolomedes plantarius*	Pisauridae	N/A	2–2.5	Wild	[13]	85, 87–88
*Dolomedes raptor*	Pisauridae	N/A	2.6	Wild	[40]	74
*Dolomedes saganus*	Pisauridae	N/A	2.4	Wild	[40]	73, 75
*Dolomedes scriptus*	Pisauridae	N/A	2.4	Wild	[27]	41, 44–46
*Dolomedes sulfureus*	Pisauridae	N/A	2.6	Captivity	[23]	
*Dolomedes tenebrosus*	Pisauridae	∼2	1.5–2.6	Wild	[27], [43]	33, 40
*Dolomedes triton*	Pisauridae	1–1.5	1.5–2.5	Wild	[20], [34], [36]	9–12, 15, 17, 19–20, 24–27, 30, 39
*Dolomedes vittatus*	Pisauridae	N/A	2	Wild	[27]	43
*Dolomedes* sp. – photographed in Queensland (not *facetus*)	Pisauridae	N/A	N/A	Wild	Loren Jarvis, pers. comm.; Robert Raven, pers. comm.	66
*Hemirrhagus pernix*	Theraphosidae	N/A	3.5	Field experiment	[25]	
*Heteropoda natans*	Sparassidae	N/A	2.5	Captivity	[21], [93]	
*Hysterocrates gigas*	Theraphosidae	N/A	4–9	Captivity	[87]	
*Nilus* spp.	Pisauridae	0.45–0.6	1.5–3	Wild	[17], [37], [39]	79–84
*Pardosa pseudoannulata*	Lycosidae	0.1	1	Wild	[28], [42], [82]	78
*Tinus* sp.	Pisauridae	N/A	1.4	Field experiment	[25]	
*Trechalea* spp. (e.g., *Trechalea tirimbina*)	Trechaleidae	∼1.5	2	Wild	[33]; Witold Lapinski, pers. comm.	48–51, 59

The genus name *Thalassius* has been changed to *Nilus* [26], [176].

* Weight roughly estimated using data for similar-sized adult female *Dolomedes tenebrosus* [43].

**Table 3:** Fresh weight and total length of fish species reported to be captured by spiders.

Fish species	Fish family	Weight (g)	Total length (cm)	Location of observation	Source	report #
*Aphosemion* spp.	Nothobranchiidae	0.2–0.7	3.5–4.5	Wild	[54], [66]	82–84
*Carassius auratus*	Cyprinidae	0.5–7	2.5–7.5	Wild	[49], [60]	11, 61–62, 65
*Carassius auratus*	Cyprinidae	∼12	9	Wild	[49]	64
*Cyphocharax* sp.	Curimatidae	N/A	6	Wild	Ed Germain, pers. comm.	52
*Cyprinus carpio*	Cyprinidae	0.2	2	Wild	Alison King, pers. comm.; Bradley Pusey, pers. comm.	89
*Elassoma zonatum*	Elassomatidae	0.1–0.5	1.7–3.1	Wild	[61]	1–8, 78
*Fundulus chrysotus*	Fundulidae	<0.1–7.8	2.3–4.5	Wild	[61]	10
*Galaxias olidus*	Galaxiidae	4–5.5	7.7	Wild	[53]	66
*Gambusia affinis*	Poeciliidae	0.5–2	3–4.5	Wild	[56]	13, 15, 17, 21–23, 34–35
*Gambusia holbrooki*	Poeciliidae	0.2–1.8	2.5–4.5	Wild	[51]–[52]	20, 28, 30, 85
*Gasteropelecus sternicla*	Gasteropelecidae	N/A	4–5	Captivity	[20]	
*Gasterosteus aculeatus*	Gasterosteidae	1.1–2.6	5.1–6.6	Captivity	[55]	
*Gila ditaenia*	Cyprinidae	4.5	N/A	Wild	[63]	9
*Glaniopsis hanitschi*	Balitoridae	0.6	∼3	Captivity	[21]; http://fishbase.mnhn.fr/PopDyn/	
*Gobiomorphus* sp.	Eleotridae	N/A	3	Captivity	[11]	
*Heterandria formosa*	Poeciliidae	<0.1–0.5	0.6–3.2	Wild	[61]	19
*Ictalurus punctatus* (fingerling)	Ictaluridae	5	6	Wild	[68]	12
*Lamprologus pulcher*	Cichlidae	N/A	1.5	Captivity	Dolores Schütz, pers. comm.	
*Lebistes* sp.	Poeciliidae	0.1	N/A	Captivity	[11], [44]	
*Lepomis cyanellus*	Centrarchidae	2	5.3	Wild	[47]	46
*Lepomis macrochirus*	Centrarchidae	0.2–3	2.7–6	Wild	[61], [67]	14
*Limia melanogaster*	Poeciliidae	N/A	3	Captivity	[22]	
*Melanotaenia* spp.	Melanotaeniidae	1	5	Wild	[58]; Bradley Pusey, pers. comm.	68
*Micropterus dolomieu*	Centrarchidae	∼1–2	4–5	Wild	http://www.garden-island.com/bass-weight-formula-calculator.htm	42
*Notemigonus crysoleucas*	Cyprinidae	1.6–2.5	6.5–7.5	Wild	[62]	40
*Oncorhynchus mykiss* (fry)	Salmonidae	1	3	Wild	[57]	32
*Oncorhynchus mykiss* (fingerling)	Salmonidae	∼3–4	5	Wild	[48]	31
*Oryzias curvinotus*	Adrianichthyidae	N/A	2–4	Wild	http://www.fishbase.org/summary/Oryzias-curvinotus.html	75
*Parazacco spilurus*	Cyprinidae	N/A	2.5	Wild	David Dudgeon, pers. comm.	76
*Phoxinus phoxinus*	Cyprinidae	0.7–2	4–6	Captivity	[1]	
*Poecilia mexicana*	Poeciliidae	0.7	2–3	Field experiment	[25], [46]	
*Poecilia reticulata*	Poeciliidae	0.5–0.8	3.5–4	Captivity	[50]	
*Pseudomugil* spp.	Pseudomugilidae	∼0.5	3.5	Wild	[58]; Bradley Pusey, pers. comm.	69
*Pseudorasbora parva*	Cyprinidae	0.7	3.3	Wild	[64]	73
*Pungitius laevis*	Gasterosteidae	0.3–0.7	3.4–4.8	Wild	[55]	87–88
*Rasbora calliura*	Cyprinidae	N/A	6–7	Wild	Michael Lo, pers. comm.	72
*Semotilus atromaculatus*	Cyprinidae	2–3	6	Wild	[45]	33
*Xiphophorus helleri*	Poeciliidae	1.3	4	Wild	[59]	67
Unknown	Order Characiformes	∼1–4	4–6	Wild	[65]	48–55, 59–60
Unknown	Percidae	N/A	N/A	Wild	[29]	44
Unknown	Pimelodidae	N/A	N/A	Wild	Craig Harrison, pers. comm.	57

Unpublished photographs of such events gained during the study were sent to ichthyologists and spider taxonomists for identification (see Acknowledgements for details). The resolution of a small number of the images was reduced sufficiently to result in uncertain identification beyond genus level but in most cases, identification to species level was considered appropriate. This is true for fish and spiders. Nomenclature of spiders follows Platnick [26]. Data on the live weight and size of spiders and fish were taken from the arachnological [11], [13], [17], [19]–[20], [22]–[25], [27]–[43] and ichthyological [1], [44]–[68] literature. Unless reported in the literature or by the respondents in our survey, the total lengths of the fish prey were estimated based on the photographs (see Table 1). The vast majority of reported spiders were adult pisaurids ∼2–2.5 cm in length (cephalothorax plus abdomen; Table 2) and knowing their approximate body length, this was used as a standard (replacing a reference scale) to roughly estimate fish lengths. In cases where spider length remained doubtful (e.g., immature *Trechalea* spp.) no estimates of fish length were made. The estimates obtained in this manner were similar to those reported in the literature where the lengths of predated fish were measured in the laboratory indicating that our estimates are fairly accurate. Report numbers used in the tables refer to the respective detailed report description (see File S1).

## Results

### Geographic Distribution of Fish Predation by Spiders

**Figure 1:**
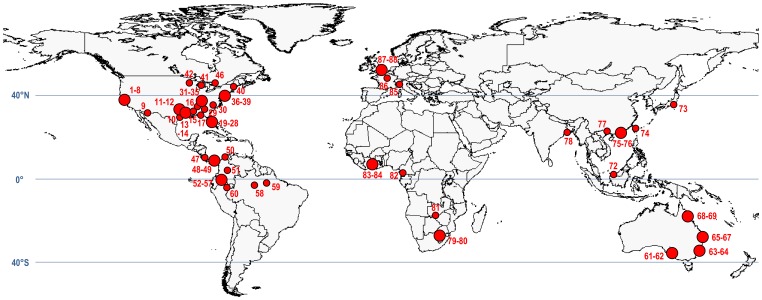
Geographic distribution of fish predation by spiders worldwide. Map depicts locations were spiders were observed predating fish (red dots). Large red dots indicate that several reports originated from same geographic region. Numbers refer to detailed report description (see File S1). GPS coordinates were unavailable for reports #18, 43–45, and 70–71; report # 89 not included.

Fish capture by spiders has been reported from all continents with the exception of Antarctica, where semi-aquatic spiders are absent ([26]; Fig. 1; Table 1). Approximately 90% of observed fish predation events were from regions of warmer climate between 40° S and 40° N (Fig. 1) and were typically observed at the margin of freshwater streams, rivers, creeks, bayous, lakes, ponds, swamps, and fens (see File S1).

**Figure 2:**
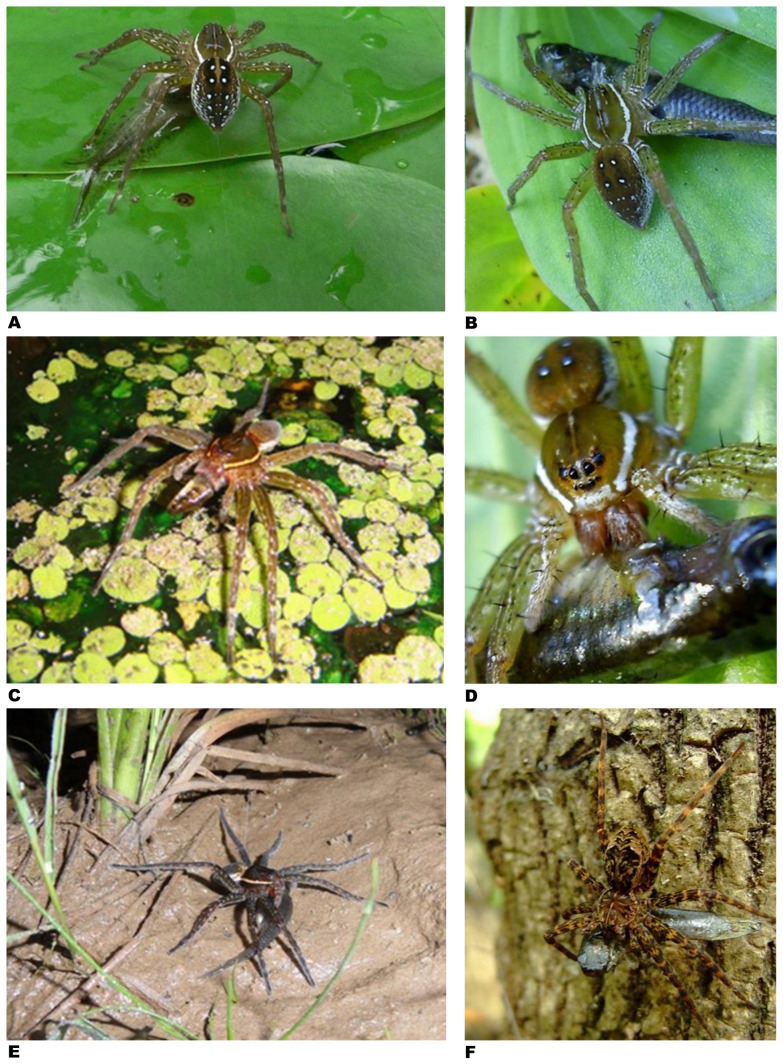
Fish caught by spiders – examples from North America. **A –** *Dolomedes triton* caught mosquitofish (*Gambusia*) in backyard pond near Tampa, Florida (photo by Stacy Cyrus, DavesGarden website; report # 24). **B –** *Dolomedes triton* feeding on fish (probably mosquitofish *Gambusia holbrooki*) in garden pond near Lady Lake, Florida (photo by Machele White, Lady Lake, Florida; report # 20). **C –** *Dolomedes triton* feeding on small fish (presumably least killifish *Heterandria formosa*) on Tsala Apopka Lake, Florida (photo by Claire Sunquist-Blunden, Ocala, Florida; report # 19). **D –** *Dolomedes triton* feeding on fish (probably mosquitofish *Gambusia holbrooki*) in garden pond near Lady Lake, Florida (same incidence as in Fig. 2B; report # 20). **E –** *Dolomedes triton* devouring fish (probably mosquitofish *Gambusia holbrooki*) on edge of small, slow-moving stream near Fayetteville, North Carolina (photo by Patrick Randall, Fort Bragg, North Carolina, USA; report # 30). **F –** *Dolomedes okefinokensis* feeding on small fish (probably mosquitofish *Gambusia holbrooki*) in swamp in Big Cypress National Preserve, Florida (photo by Misti Little, Stagecoach, Texas; report # 28).

**Figure 3:**
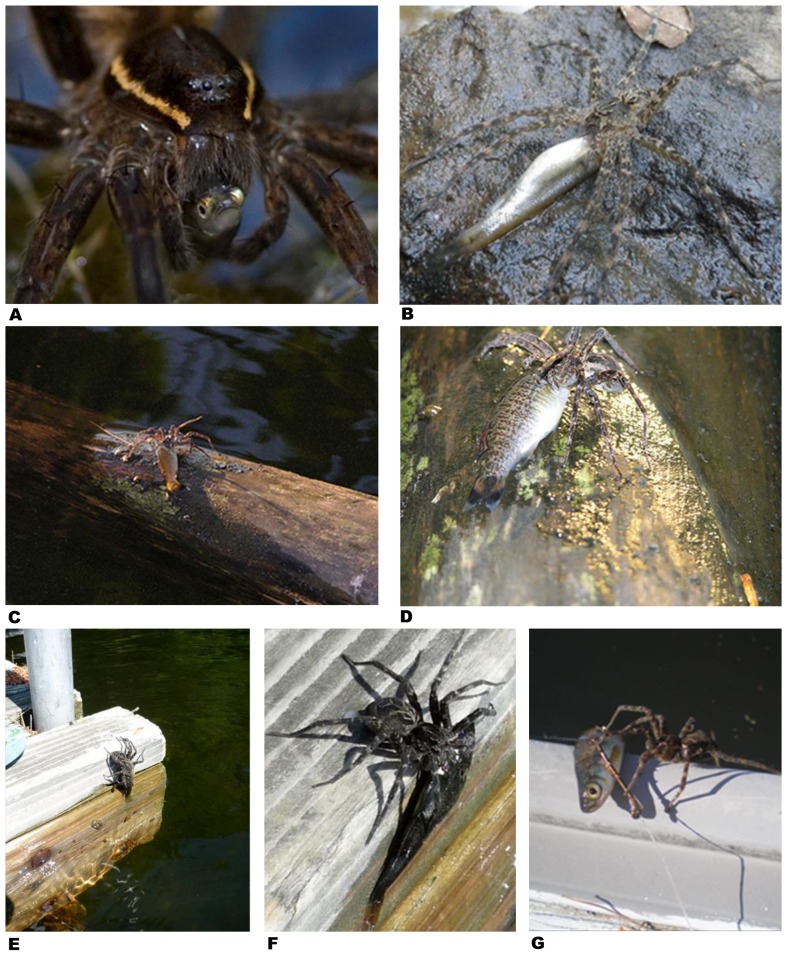
Fish caught by spiders – examples from North America. **A –** *Dolomedes triton* captured Sonora chub (*Gila ditaenia*) in small stream in Sycamore Canyon, Pajarito Mountains, Arizona (photo by Andreas Kettenburg, Thousand Oaks, California, USA; report # 9). **B –** *Dolomedes tenebrosus* devouring creek chub (*Semotilus atromaculatus*) on bank of Bullskin Creek near Brutus, Kentucky (photo by Jason Butler, Lexington, USA; report # 33). **C, D –** *Dolomedes* sp. caught smallmouth bass, *Micropterus dolomieu*, on shore of Flambeau River near Ladysmith, Wisconsin, USA (photo by Tod Lewis, Austin, Texas; report # 42). **E, F –** *Dolomedes scriptus* feeding on fish (probably green sunfish *Lepomis cyanellus*) fished out of 1.8 m deep water on dock at Shoe Lake near Dorset, Ontario, Canada (photo by Lloyd Alter, Toronto, Canada; report # 46). **G –** *Dolomedes* sp. scuttled out very quickly from underneath dock attempting to attack live bait fish (probably golden shiner *Notemigonus crysoleucas)* after a mis-cast resulted in bait fish landing just off edge of dock near Sebago Lake, Maine, USA (photo by Jeffrey Hollis, East Haddam, Connecticut, USA; report # 40).

Fish predation by spiders has been most frequently documented in North America, with 45 incidences from the USA (51% of the total; Figs. 2–3; Table 1; report # 1–45) with those concentrated in the east and southeast, particularly in Florida wetlands and neighbouring regions (Fig. 2). Elsewhere in North America, nine incidences of fish predation from the western USA are known to us (eight from California and one from Arizona; report # 1–9), two from the Midwest (one from Michigan and one from Wisconsin; report # 41–42) and a single observation from Canada of a semi-aquatic spider feeding on a fish while sitting on the dock wall at Shoe Lake, Ontario (report # 46).

**Figure 4:**
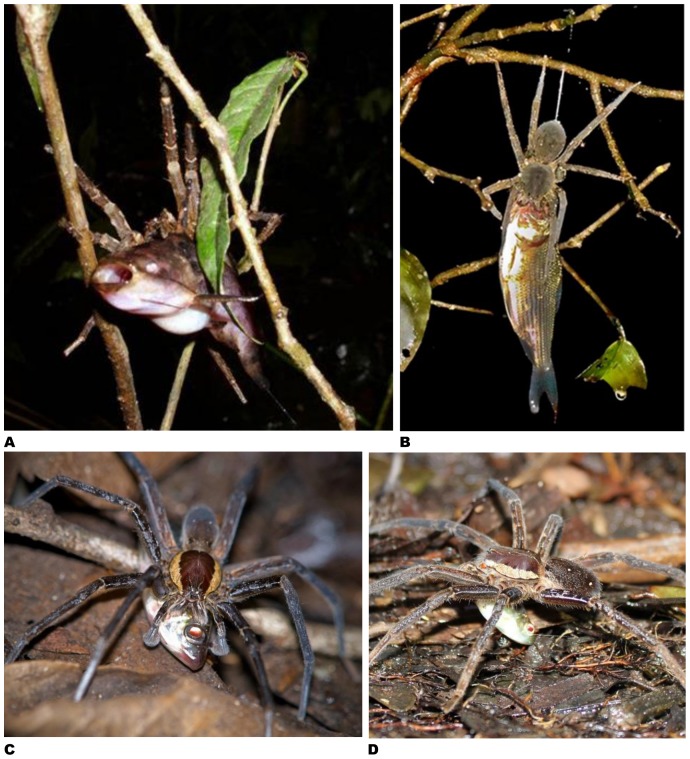
Fish caught by spiders – examples from the Neotropics. **A –** In marshy area in Cuyabeno Wildlife Reserve, Ecuador, adult *Ancylometes* sp. (probably *Ancylometes rufus*) lifted catfish - most likely family Pimelodidae - out of the water (photo by Craig Harrison, Hertford, UK; report # 57). **B –** Ctenid spider (*Ancylometes* sp.) feeding on characiform in Tahuayo river area de Conservacion Regional Communal Tamshiyacu-Tahuayo Loreto, Peru (photo by Alfredo Dosantos Santillan c/o Amazonia Expeditions, Tampa, USA; report # 60). **C –** Adult male of *Ancylometes* sp. (possibly *Ancylometes rufus*) caught characiform (*Cyphocharax* sp.) near Samona Lodge, Cuyabeno Wildlife Reserve, Ecuador (photo by Ed Germain, Sydney, Australia; report # 52). **D –** Adult *Ancylometes* sp. preying on characiform near Samona Lodge, Cuyabeno Wildlife Reserve, Ecuador (photo by Tim Wohlberg, Kelowna, BC Canada; report # 53).

**Figure 5 pone-0099459-g005:**
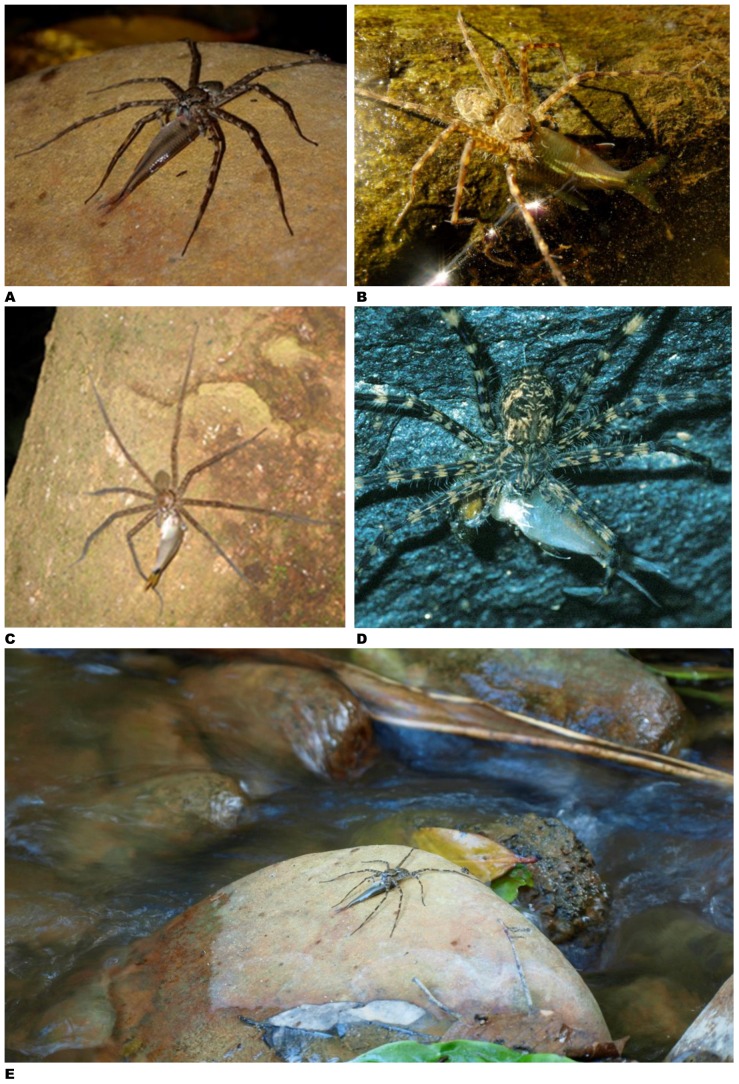
Fish caught by spiders – examples from the Neotropics. **A –** *Trechalea* sp. eating characiform while sitting on a rock in middle of small river near Paratebueno, Cundinamarca, Colombia (photo by Solimary Garcia Hernandez, Instituto de Biociências, Universidade de São Paulo, Brazil; report # 51). **B –** *Trechalea* sp. pulling characiform on stone on edge of shallow, small stream near Quebrada Valencia, Magdalena, Colombia (photo by Juan Esteban Arias A., Cali, Colombia; report # 50). **C –** *Trechalea* sp. devouring characiform while sitting on tree trunk on edge of Rio Frijoles, Central Panama (photo by Jessica Stapley, University of Sheffield, UK; report # 48). **D –** *Trechalea* sp. eating characiform on bank of Rio Maicuru, Pará State, Brazil (photo by Jacques Jangoux, Belém, Brazil; report # 59). **E –** *Trechalea* sp. eating characiform while sitting on rock in middle of small river near Paratebueno, Cundinamarca, Colombia (photo by Solimary Garcia Hernandez, Instituto de Biociências, Universidade de São Paulo, Brazil; report # 51).

**Figure 6 pone-0099459-g006:**
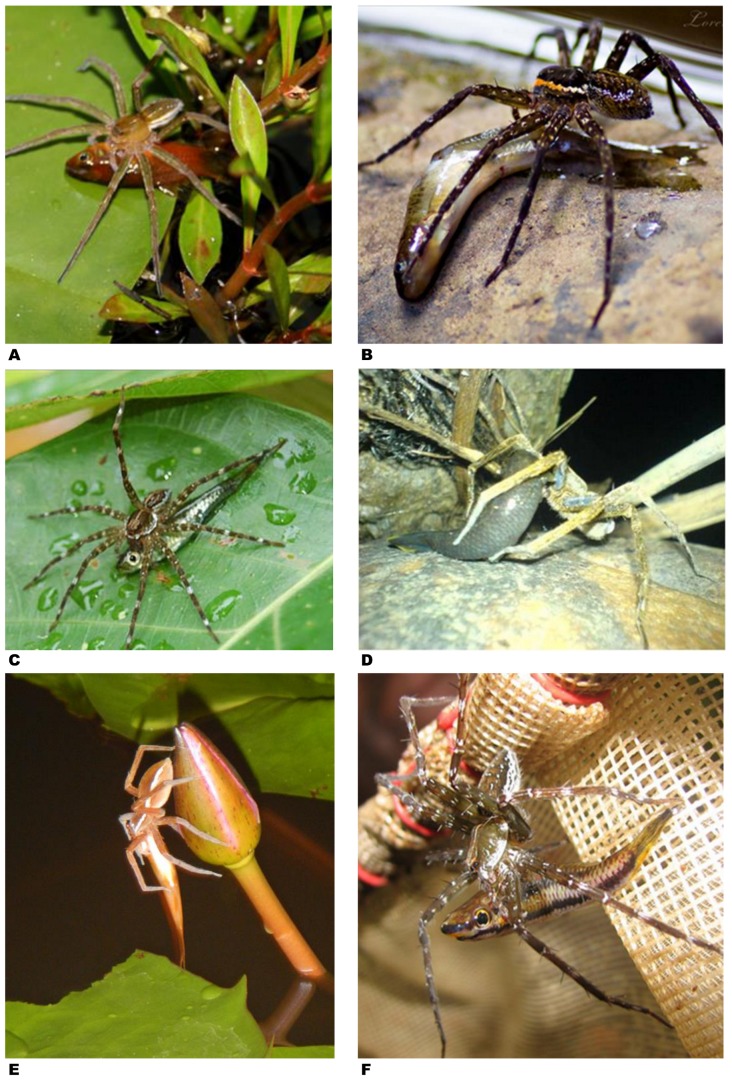
Fish caught by spiders – examples from Australia, Asia, and Africa. **A –** *Dolomedes facetus* captured pond fish (genus *Xiphophorus*) in garden pond near Brisbane, Queensland, Australia (photo by Peter Liley, Moffat Beach, Queensland; report # 67). **B –** *Dolomedes* sp. preying on mountain galaxias (*Galaxias olidus*) on bank of North Branch Creek near Goomburra, Queensland, Australia (photo by Loren Jarvis, near Brisbane, Queensland; report # 66). **C –** Semi-aquatic pisaurid devouring fish (presumably *Rasbora calliura*) at edge of shallow river flowing through forest in eastern Batang Sadong basin, Borneo (photo by Michael Lo, City of Kuching, Malaysia; report # 72). **D –** Unspecified teleost fish captured by *Dolomedes raptor* on edge of stream near Tung-Shih, Taichung county, Taiwan (photo by Tai-Shen Lin, Tunghai University, Taiwan; report # 74). **E –** Semi-aquatic pisaurid spider (*Nilus* sp.), dangling from lily flower bud, pulled unidentified fish (∼4 cm in length) out of water of garden pond in Victoria Falls, Zimbabwe (photo by Marcelo de Freitas, Cresta, South Africa; report # 81). **F –** Pisaurid spider (*Nilus* sp.) in fish net attacked and captured small killifish (*Aphyosemion* sp.) in stream near city of Kribi, Cameroon (photo by Duncan Reid, Yale University, USA; report # 82).

**Figure 7:**
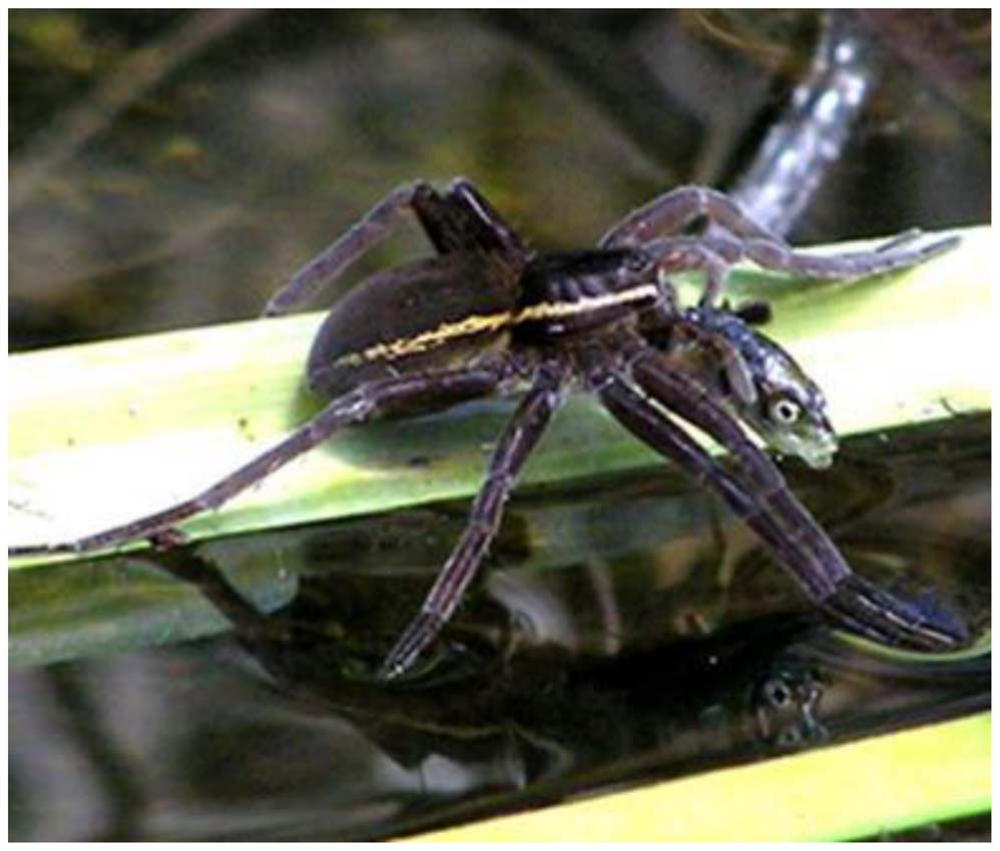
Fish caught by spider – example from Europe. Gravid adult female *Dolomedes plantarius* captured stickleback (*Pungitius laevis*) in turf pond at Redgrave and Lopham Fen National Nature Reserve in East Anglia, UK (photo by Helen Smith, South Lopham, Norfolk, UK; report # 87).

Multiple incidences of fish predation have been reported (14 reports) from the Neotropics of large semi-aquatic spiders with a nocturnal life-style found either on the banks of rivers and streams in tropical forests in Brazil, Colombia, Costa Rica, Panama, and Peru or near shallow puddles and creeks on the lowland tropical forest floor in Ecuador (Figs. 4-5; report # 47–60). A third region where multiple fish predation events have been witnessed is Australia (twelve incidences; report # 61–71, 89), where pond fish were repeatedly caught by spiders in suburban/urban gardens of Adelaide, Brisbane, Lismore and Sydney or native freshwater fish were predated by spiders on the fringes of slow flowing streams in New South Wales and Queensland ([19]; Loren Jarvis, pers. comm.; Bradley Pusey, pers. comm.; Figs. 6A–B). Less common are reports of predation in Asia (seven reports, Figs. 6C–D; report # 72–78), which is surprising given the richness of spider taxa throughout this region [26], [42]. Similarly, there is a paucity of information on fish predation by spiders in Africa, with only six documented cases from tropical secondary forests and garden ponds (Fig. 6E–F; report # 79–84). Interestingly, only four reports originate in Europe, namely from the United Kingdom, Italy, and France (Fig. 7; Table 1; report # 85–88).

### Which Spider Species are Engaged in Fish Predation?

**Figure 8:**
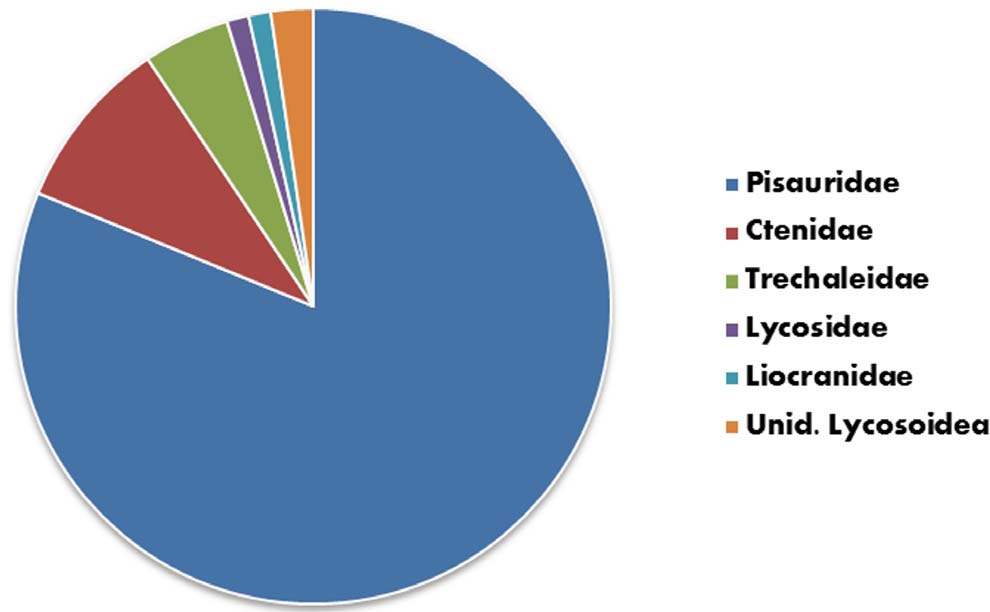
Relative importance of different spider families as fish predators – based on 89 incidences reported in Table 1.

The superfamilies Lycosoidea and Ctenoidea are those documented as preying upon fish under open-field conditions and all can be loosely categorized as hunting spiders (i.e., spiders that forage without the use of a catching web). Approximately 80% of reports of fish predation were attributable to Pisauridae (nursery web spiders), with Ctenidae (wandering spiders; 10.3%), Trechaleidae (longlegged water spiders; 4.5%), Lycosidae (wolf spiders; 1.1%) and Liocranidae (spinylegged sac spiders; 1.1%) comprising the remainder (Fig. 8; Table 1).

The most dominant group of fish-catching spiders are in the genus *Dolomedes* (Pisauridae) (Figs. 2–3, 6A–B, D, 7-8). The spiders in this worldwide distributed genus are semi-aquatic predators with a legspan of 6–9 cm and a weight of ∼0.5–2 g ([26]; Table 2). Although the various *Dolomedes* spp. appear to differ in their foraging time, some species being diurnal and others nocturnal (File S1; [11]), these spiders share a common foraging trait in that they can swim, dive and walk on the water's surface film [69]. Indeed, the spiders in this genus are voracious predators with broad diets [69]–[71] and of the more than 90 species in *Dolomedes* (see Platnick [26]), eleven have been recorded catching fish in the wild (Figs. 2–3, 6A–B, D, 7; Tables 1–2). A species particularly adept at catching and eating fish in the wild is the North American *Dolomedes triton* (Figs. 2A–E, 3A; Table 1). Three more species in this genus have been observed catching fish under laboratory conditions (Table 2) suggesting that the real number of fish-catching *Dolomedes* is considerably higher than the number reported in this paper. Some species may, however, be poorly adapted to catching fish. For example, only the largest of New Zealand's three species of *Dolomedes* (*Dolomedes dondalei*) was capable of catching fish in laboratory experiments whereas the two smaller species (*Dolomedes aquaticus* and *Dolomedes minor*) were not [11].

Elsewhere in the Pisauridae, the spiders of the genus *Nilus* (Figs. 6E–F) are also semi-aquatic with similar feeding habits as *Dolomedes* [72]. They have a legspan of ∼6 cm and a weight of ∼0.5 g (Table 2). This genus is restricted to Africa and Asia [26] and thus far only two species – *Nilus curtus* and *Nilus massajae* – have been reported as engaging in fish predation under natural conditions (Fig. 6; Table 1). Given that other pisaurid spiders have a similar semi-aquatic lifestyle (e.g., *Megadolomedes, Thaumasia*, and *Tinus*), it can reasonably be deduced that such species could include fish in their diet as well ([25], [73]; Kelly Swing, pers. comm.).

A number of spiders outside the Pisauridae also prey upon fish. Spiders in the genus *Ancylometes* (Ctenidae) (Fig. 4) occur mostly in South America, typically inhabiting moist Neotropical forests where they hunt at night at the edge of water bodies [26], [35]. *Ancylometes rufus*, the largest species in this genus, has a leg span of 20 cm and a weight of up to 7 g [35]. *Ancylometes* can dive for up to 20 minutes [35] and at least two species – *Ancylometes bogotensis* and *Ancylometes rufus* – are known to catch fish in the wild (Tables 1–2). In addition to feeding on fish, these spiders also predate a variety of other small vertebrates such as tadpoles, frogs, toads, and lizards [41], [74]–[77].

Members of the Neotropical genus *Trechalea* (Trechaleidae) also engage in fish predation ([17]; Fig. 5). These spiders are also large (average adult weight of ∼1.5 g and a legspan of up to 17 cm) and are typically found near shallow freshwater streams in Central and South America [33], [78]–[79]. Adults are strictly nocturnal whereas immatures frequently hunt both day and night [33], [78]–[79]. The trechaleids depicted in Fig. 5 could not be identified to species and might be immatures given that they were photographed during the daylight hours. Contrary to the pisaurids, the trechaleids do not chase their prey across the water surface and unlike many other fish-catching spiders are not capable of diving [79]. In similar fashion to the ctenids, *Trechalea* spp. feed on a diverse array of other food items, including insects, shrimps, and frogs [78]–[80].

Aside from the abovementioned spiders, with well documented though poorly understood fish-catching abilities, a number of other spiders have been reported in the literature as occasionally preying upon fish (Table 2). For example, the diurnal *Pardosa pseudoannulata* (pond wolf spider) is limited to parts of Asia and is found in stagnant pools, rice fields and swamps where it rests on aquatic plants [26], [81]–[82]. This semi-aquatic spider, capable of swimming and diving, is one of the smallest spiders observed catching fish and there is only one report of such an event witnessed in the wild from a pond in India [82]. However, the fish-catching capability of *Pardosa pseudoannulata* has further been documented in the laboratory [82]. The capture and consumption of small vertebrates other than fish by lycosids, however, has repeatedly been reported in the literature [7], [16], [41], [76], [83]–[86]. This suggests that many other lycosids could include fish in their diet as well.

There is laboratory evidence that spider species of even more families (e.g., Cybaeidae, Desidae, and Sparassidae) are capable of catching and eating fish [19], [21], [25], [85], [87]. A good example is the water spider *Argyroneta aquatica* (Cybaeidae), the only truly aquatic spider (spending its entire life under water) known so far, which has been observed killing and devouring tiny fish of the families Cichlidae, Gasterosteidae, and Poeciliidae when kept in aquaria ([23], [38], [85], [88]–[90]; Dolores Schütz, pers. comm.). This small spider (∼0.1–0.3 g), equipped with very potent venom enabling it to kill tiny fish instantly, constructs a ‘diving bell’ (i.e., dome-shaped underwater web filled with air) between aquatic plants in which it digests prey, mates, etc. [23], [38], [90]. Two other examples of spiders reported catching fish in captivity are the semi-marine species *Desis marina* (Desidae) and the ‘swimming’ huntsman spider *Heteropoda natans* (Sparassidae) [21], [91]–[93]. There are also reports of tarantulas (Theraphosidae) swimming and diving in the laboratory [87] and there are even a few reports of tarantulas seen swimming in the wild [94]. Captive theraphosids devoured dead freshwater fish which is not surprising given that these spiders are voracious, opportunistic feeders [95]–[96]. Some researchers assumed that certain theraphosid species in the subfamilies Eumenophorinae and Theraphosinae might be capable of predating fish [25], [87]; but there are others who consider this as improbable (Robert Raven, pers. comm.).

### How Do Spiders Catch Fish?

The prey capture and feeding behavior of *Dolomedes* and *Nilus* has been well documented [11], [16], [20], [69], [72], [97]–[98] and it was widely reported in the past that these semi-aquatic spiders depend largely on vision for prey detection [69]. However, it has since become apparent that vision plays a relatively minor role in prey detection and instead both *Dolomedes* and *Nilus* rely on stimuli perceived by mechanoreception [11], [20]. Typically, semi-aquatic pisaurids anchor their hind legs to a stone or plant, with their front legs resting on the surface of the water [11], [19], [29], [72], [98], ready to ambush their prey. Carico [29] states: “….they use the surface film as if it were a web, because they sit at the edge of the water and pursue insects that accidentially fall upon the water and are trapped by the surface tension”. He continues “….Apparently the ripples caused by an insect provide the stimulus for the predatory response and possibly also provide information as to the location of the prey”. While this behavior may be efficient for catching insect prey, it may be less effective at catching fish due to the spiders' rather low response to fish-generated surface waves [20] and only works under calm conditions [11]. In laboratory experiments the majority of successful fish catches occurred after the spiders' attack behavior had been triggered through direct touch, facilitated by the dorsal fin of a fish touching one of the spider's outstretched legs [11], [20]. The fish catching behavior of ctenids and trechaleids resembles that of the pisaurids [14], [22], [33], [78]–[79].

### Which Species of Fishes are Captured by Spiders?

All 89 cases of fish predation listed in Table 1 involve small freshwater fish. In 17% of the reported incidences the fish prey remained unidentified. The identifiable prey belonged primarily to the orders Cyprinodontiformes (28% of total identifiable fish prey), Cypriniformes (22%), Perciformes (20%), and Characiformes (14%) but also included the Atheriniformes, Beloniformes, Gasterosteiformes, Osmeriformes, Salmoniformes, and Siluriformes (Table 3). The captured fish usually are among the most common fish occurring in their respective geographic area (e.g., mosquitofish [*Gambusia* spp.] in the southeastern USA, fish of the order Characiformes in the Neotropics, killifish [*Aphyosemion* spp.] in Central and West Africa, as well as Australian fishes of the genera *Galaxias*, *Melanotaenia*, and *Pseudomugil*). Spiders killed small-sized fish predominantly 2–6 cm in length (Table 1; File S1). Prey species included adults of small bodied species, usually weighing ∼0.1–7 g (Table 3; e.g., *Aphyosemion* spp., *Elassoma zonatum*, *Gambusia* spp., *Heterandria formosa,* and *Pungitius laevis*) or the very small immatures of species which achieve larger body size (e.g., *Ictalurus punctatus, Lepomis cyanellus*, *Lepomis macrochirus*, *Micropterus dolomieu*, and *Oncorhynchus mykiss*). *Ictalurus punctatus* and *Oncorhynchus mykiss* can reach a body weight of >10 kg once fully grown.

### Predator–Prey Size Ratio

In general, spiders feed predominantly on prey items – usually insects – that are smaller than themselves [99]. This is true in spiders from many different families including semi-aquatic pisaurid spiders feeding on insect prey [100]–[101]. However, results herein show that semi-aquatic spiders from different families captured fish prey whose body length exceeded the spiders' body length (the captured fish being, on average, 2.2 times as long as the spiders [based on data from Table 1]).

Similar departures involve weight; spiders of the genera *Dolomedes* and *Nilus,* with a weight of ∼0.5–2 g (Table 2), can catch fish prey up to 4.5 times the spider's weight [15]–[16], [20]. In laboratory experiments, attempts by *Dolomedes triton* to catch goldfish, *Carassius auratus*, weighing 7.5–10.5 times the spiders' weight always failed (Bleckmann & Lotz [20]). Under the assumption that the largest fishing spider, the ctenid *Ancylometes rufus* weighing up to 7 g (Table 2), is as effective in overpowering oversized prey as the smaller-sized pisaurids, fish of up to 30 g might conceivably be killed in the wild. The largest fish reported, however, to have been captured by a pisaurid spider was a *Carassius auratus* ∼9 cm in length and presumably weighing >10 g (Table 3); this incidence had been witnessed in a garden pond in Sydney, Australia (report # 64).

## Discussion

### Are the Documented Incidences of Fish Consumption Real Predation Events?

It is arguable whether all incidences reported in this paper are real predation events or whether some are just cases of scavenging. Predation requires that a prey item must have been killed and eaten by the predator [102]. Both behavioral traits – killing and consumption – have been witnessed many times by a large number of researchers in the wild and in captivity. These spiders possess large strong chelicerae capable of piercing the skin of vertebrates [23] and are equipped with powerful venoms containing hundreds of different neurotoxins, some of which are specific to vertebrate nervous systems [103]–[105]. The vast majority of fish (∼85%) are bitten by the spiders at the base of the head (Figs. 2–7). How long it takes in a particular case to kill a fish depends on the size and species of the fish in question [23]. Small fish with a thin skin may die within a few seconds to minutes after the bite [13], [19]–[20], [23]–[24], [69] although burbot fish (*Lota lota*) with an average fresh weight of ∼0.4 g required 50 minutes before death [23]. Injecton of the venom of the semi-aquatic spider *Dolomedes sulfureus* into the thorax of zebrafish (*Danio rerio*) in the laboratory caused severe neurological disturbance resulting in disorientation, uncoordinated movement (spinning), lack of buoyancy control and ultimately death within 20 min [24]. Gudger [15] reports two cases, both witnessed in the wild, where fish bitten by *Dolomedes* spiders exhibited similar spinning behavior prior to dying.

A fish prey must always first be dragged by the spider to a dry place before the feeding process can begin [13]. Such a dry feeding site can be a rock, tree trunk, halfway immersed log, or an aquatic plant emerging from the water (Figs. 2A–B, D–F, 3B–G, 4–7). The behavior of always first moving a fish prey to a dry site prior to feeding can be explained by the spiders' extraintestinal digestion – first pumping digestive enzimes into the prey and thereafter sucking in the dissolved tissue through the mouth opening [106]; otherwise the digestive enzimes would be diluted in the water and, thus, become ineffective [8]. This type of feeding behavior has been witnessed in spiders from all families engaged in fish-catching. A second reason for this behavior may be that on land the spider has physical superiority over its aquatic prey and its potential for escape is greatly reduced.

In captivity *Dolomedes* spiders accepted dead sticklebacks as food, but this was observed only in hungry adult females during periods of increased food requirements between mating and oviposition [69]. Thus, scavenging may occasionally occur in the wild as well, if the spiders are hungry enough. On the other hand, *Trechalea* spp. did not feed on dead prey if offered in captivity [79], [107]. A special case is given when spiders grab fish in fishing nets (Fig. 6F; report # 82) or – as in one case observed in Maine – attack a live bait fish landing on the dock wall after a fishing mis-cast (Fig. 3G; report # 40). We consider such incidences as predation attempts since the spider is grabbing a living fish with the intention to kill and devour it. The fact that fish are attacked even outside the water shows the high propensity for such spiders to feed on fish. A careful consideration of all the evidence available to us, where spiders had been observed/photographed feeding on fish in the wild and in captivity, led to the conclusion that the vast majority of incidences reported in this review refer to fish predation and not scavenging.

### How Frequent are Incidences of Fish Predation by Spiders?

The majority of incidences of spider predation upon fishes were reported from the Americas, especially the eastern part of USA, whereas few were reported from Africa, Asia, Australia or Europe. To a large extent, the pattern shown in Fig. 1 may simply reflect the distribution of potential observers and especially those with the capacity or propensity to report observations of spider predation. There is a high concentration of major universities and government agencies with research labs engaged in ecological projects in nearby wetland habitats in the eastern part of USA. In addition, this region contains numerous nature enthusiasts that visit the wetlands of the eastern USA for recreation and subsequently post reports and photographs of rare incidences on the world wide web. Such a concentration of researchers and enthusiasts is unlikely elsewhere, with the exception of Europe which plausibly contains as many research institutions, scientists and amateur enthusiasts, and thus may potentially bias our view of geographical patterns of the incidence of spider predation on fish. In the Neotropical region, fish predation was probably strongly underreported due to the fact that the dominant Neotropical semi-aquatic spiders (i.e., *Trechalea* spp. and *Ancylometes* spp.) occur most commonly in remote areas of the tropical rainforest and are strictly nocturnal as adults [22], [35], [78]–[79], characteristics that make their observation in the wild difficult.

Semi-aquatic spiders may be more common in some geographic regions than in others and our reported distribution of observations may reflect this difference in abundance and diversity. Semi-aquatic spiders are very common in eastern USA, particularly in the freshwater wetlands of Florida and neighboring regions (6 spp.; [29]). Nine species of *Dolomedes* occur in North America, in contrast to Europe which contains only two species [13], [29]. One of the two European species (*Dolomedes plantarius*; Fig. 7), which is associated with open water and which is known to predate upon fish in the wild, has now become so rare that it is considered a threatened species – largely a consequence of the continuous loss of European freshwater wetland habitat [13], [108]–[110]. The second European species (*Dolomedes fimbriatus*) appears to depend on open water to a lesser degree [13], [108], [111] and has so far never been seen predating fish in the wild (e.g., Heiko Bellmann, pers. comm.; Emanuele Biggi, pers. comm.; Franz Renner, pers. comm.; Jakob Walter, pers. comm.). It must be said that *Dolomedes fimbriatus* often occurs in wetlands such as highly acidic moorlands or seasonally intermittent marshy areas in which fish are naturally absent [101], [111]. *Dolomedes fimbriatus* is perhaps less well-adapted to predating fish in the wild. An extensive observational study by Poppe & Holl [101] in moorlands of northwestern Germany revealed that *Dolomedes fimbriatus* very rarely exhibited a behavior of ‘fishing’ or underwater hunting while foraging for aquatic arthropod prey. Instead this species fed predominantly on terrestrial arthropods captured on plants [101]. Thus, it appears that spiders of the genus *Dolomedes* are less common (or as in the case of *Dolomedes fimbriatus* show less affinity with water inhabited by fish) in Europe than in the eastern part of USA. This may result in a lower likelihood of encountering or observing feeding on fish prey by such spiders in Europe. Notably however, *Dolomedes* is particularly species rich in south-east Asia [26], yet we found few verifiable records of fish predation for this region; more likely due to underreporting than the absence of fish predation per se.

Most of the reported incidences of fish predation were from a broad latitudinal band from 40° S to 40° N, with very few reported instances of fish predation by spiders occurring north of 40° N despite the fact that members of *Dolomedes*, at least, occur there [29]. This is true in both North America and Europe. The difference between the frequency of fish predation at high versus low latitude in North America is well illustrated if we compare a study from a northern location (latitude 56° N, Fairview, Canada) with one from a southern location (latitude 27° N, Tampa Bay area, Florida). Spending approximately 400 man-hours conducting some 13,000 field observations while wading slowly at the perimeter of natural ponds near Fairview, Zimmermann & Spence [100] never witnessed incidences of fish predation by *Dolomedes triton* (from a total of 625 predation events). Instead the spiders were seen feeding almost exclusively on arthropods and in one instance on a small frog [100]. The rarity of fish predation in Canada is also evidenced by the fact that according to our knowledge only one such incidence has so far been reported in this country, and this refers to a location in the most southern part of Ontario (latitude 45° N; Lloyd Alter, pers. comm.). In contrast, Brian Kenney (pers. comm.) witnessed at least half a dozen incidences of fish predation by *Dolomedes triton* while spending approximately 300 man-hours as a wildlife photographer in freshwater wetlands in the Tampa Bay area in Florida.

Fish eating spiders are constrained in the size of fish they can capture and a greater reliance on fish as prey may occur in regions inhabited by more small-bodied fish, not withstanding the fact that all large-bodied fish must also be small-bodied while juvenile. Freshwater fish assemblages of North America and Europe do indeed contain fewer small-bodied species at higher latitudes [112]–[114]. Further support of the hypothesis that the availability of fish of a suitable size increases reliance on this food source (assuming that reliance may be related to the frequency with which instances of fish predating upon fish are observed and reported) is provided by the relative reporting differences for eastern and western North America shown in Fig. 1. Moyle & Herbold [115] report that the comparatively higher richness observed in eastern North American rivers compared to western North America is mainly due to the highly diversified, small-bodied taxa that occur principally in small streams in the former region.

Reduced oxygen levels lead to higher risk of predation for fish, since the fish tend to rise to the surface to exploit the oxygen-saturated surface layer [116]–[118]. Depletion of dissolved oxygen is particularly severe in heavily vegetated swamps and stagnant pools such as those that occur in Florida and neighboring regions [119]. Areas located at higher latitudes like Canada or much of Europe are likely characterized by lower temperatures coupled with comparatively higher dissolved oxygen levels [120], potentially resulting in lower risk of predation by *Dolomedes* spp. The same may be true for freshwater wetlands in the northern part of Asia where very few incidences of fish predation have been reported so far (Fig. 1). In Florida and neigboring regions, where fish predation has been particularly frequently witnessed, the captured fish were often mosquitofish (*Gambusia* spp.) (Figs. 2A–B, D–F). These fish, which are morphologically and behaviorally well-adapted for inhabiting oxygen deficient waters [116], are among the most abundant fish in the wetlands of this geographic region [119], [121]–[122] and with a live weight of ∼0.1–1.5 g (Table 3) they optimally fit the spiders' prey size range. As ‘surface feeders’, feeding on insects trapped at the water surface, and as ‘surface breathers’ these fish are in particular vulnerable to the attack by *Dolomedes* spp. which can walk and run on the water surface [123]. Foraging at the water's surface for terrestrial arthropod prey by both predator and prey (spider and fish, respectively) may also increase the likelihood of interaction for a range of fish species other than *Gambusia*. Australian rainbowfish (*Melanotaenia*) and blue-eyes *(Pseudomugil*) both consume terrestrial insects from the water surface [58] and both have been observed being consumed by spiders (Bradley Pusey, pers. comm.; Table 1). It is striking that the geographic distribution of fish predation by spiders in North America (Fig. 1) overlaps largely with that of mosquitofish [124]. Mosquitofish occur also in streams of southern Europe (i.e., certain regions in Portugal, Spain, southern France, Italy, and Greece; [125]) and one of the four incidences of fish predation witnessed in Europe involved this species (report # 85). While extensive ecological field studies had been conducted in the more northern parts of Europe, the ecology of spiders in southern Europe is less well-studied and it is possible that fish predation by spiders in that region has been underreported due to the lack of studies conducted in mosquitofish habitats. In large parts of Europe and Palearctic Asia, mosquitofish are absent and *Dolomedes* spiders may have difficulty catching other types of resident small fish, all of which are less likely to venture close to the water surface (e.g., sticklebacks and minnows of the genus *Phoxinus*) (David Dudgeon, pers. comm.). This seems to be in line with the observations of filmmaker Martin Dohrn (Bristol, UK), whereupon several specimens of *Dolomedes fimbriatus* had difficulty capturing sticklebacks (*Gasterosteus aculeatus*) and minnows (*Phoxinus phoxinus*) in a pool (staged situation). In the case of the sticklebacks, their dorsal spines makes it difficult for predators to catch them [126] and for spiders to bite them at the base of their head. Nevertheless, sticklebacks have very rarely been captured by *Dolomedes plantarius* in a turf pond at Redgrave and Lopham Fen National Nature Reserve in East Anglia, UK (Fig. 7; report # 87–88). Other factors may also drive habitat selection by fish and increase their likelihood of encountering a waiting spider. For example, *Gambusia* spp. frequently occur in shallow water or densely vegetated stream margins in order to avoid predation by piscivorous fishes, a behavior common amongst small-bodied stream dwelling fishes [127].

### How Important is Fish Predation in Nutritional Ecology of Spiders?

All five spider families reported in this paper as fish predators under natural conditions (Ctenidae, Liocranidae, Lycosidae, Pisauridae, and Trechaleidae) are known from the literature to feed predominantly on arthropods [79], [100]–[101], [128]–[130]. The feeding biology of ctenids, liocranids, lycosids, and trechaleids in the field is still poorly understood and one cannot currently judge whether predating fish is significant from a feeding ecological point of view. However, the one incidence of a spider of the family Liocranidae feeding on a tiny fish is rather surprising. Due to its terrestrial life style and its very small size (measuring <1 cm in length) this liocranid is an unlikely fish predator and the incidence reported here (report # 86) might have been a peculiar chance event.

Fish probably constitute a minor proportion of the diet of semi-aquatic members of the family Pisauridae [8], [11], [100]–[101]. Nonetheless, in certain circumstances predation may become highly focussed upon fish. For example, fish predation by semi-aquatic pisaurids has been particularly frequently witnessed in shallow freshwater wetlands at many different locations in Florida (report # 17–28), with multiple incidences of this feeding behavior witnessed at a single location (e.g., [121]). Gudger [17] and Meehean [131] noted that large numbers of small fish were killed and devoured by semi-aquatic pisaurids in hatchery rearing ponds in Oklahoma and Tennessee, respectively (report # 12, 31–32). In one rearing pond in Oklahoma the spiders exhibited a behavior of ‘wasteful killing’ of fish (i.e., despite apparent satiation the spiders continued killing fish, thereafter consuming each fish prey item only partially; see [131]–[133]). Semi-aquatic pisaurids are indeed capable of killing several prey in succession [23]. Still another example of piscivorous feeding behavior is reported from the San Francisco, California, area where numerous small fish were killed and devoured within just a few days by a single spider specimen (presumably a *Dolomedes* sp.), that had become established next to an aquarium in the Steinhart Aquarium building ([18]; report # 1–8). Admittedly, these examples are not based on natural conditions but clearly the spiders involved evidently were temporarily specializing on fish prey (i.e., complete piscivory). This suggests that although *Dolomedes* spp. are predators with broad diets composed of invertebrates and vertebrates [69]–[71], they are capable of temporarily narrowing their feeding niche by feeding to a large extent on small fish prey when this prey type becomes available in large numbers.

Multiple incidences of fish predation by semi-aquatic spiders have been reported from the Cuyabeno Wildlife Reserve in Ecuador (report # 52–57). In this wildlife reserve, semi-aquatic ctenid spiders (*Ancylometes* spp.) were observed to feed heavily on fish at the edge of forest puddles or creeks. There is substantial observational evidence that subadults and adults of *Ancylometes* spp. feed to a large extent on small aquatic vertebrates [35], [41], [74]–[77], [134]–[137], whereas the immatures of these spider species are probably predominantly arthropod eaters ([129]; Thierry Gasnier, pers. comm.). In this case, the relative reward of switching to a diet comprised substantially of fish may have been improved by more efficient foraging due to the shallow nature of the aquatic habitat (i.e., puddles).

It takes semi-aquatic spiders many hours to consume a fish [11], [13], [69], suggesting that spiders can extract a substantial amount of energy while feeding on such large prey. Indeed, a fish prey has a ∼20–200 times higher biomass than average-sized insect prey (e.g., water striders *Gerris* spp. with an average weight of ∼0.03 g; Table 4). Typically predators are much larger than their prey. Brose et al. [138] estimate an average log_10_ predator/prey ratio of 1.62±0.03 (i.e., 42 times larger) and in this respect, the disparity in size of fish catching spiders and their prey is especially notworthy. It must be added that the caloric value of fish and insect tissue do not differ significantly (∼20–24 kJ/g dry weight; Table 4) and that fish and insect prey are both excellent sources of protein [139]. A substantial proportion of the mass of an arthropod is comprised of exoskeleton which is of no nutritional value to a spider. In contrast, the great bulk of the mass of a fish is comprised of muscle tissue. On an individual prey basis, fish are likely a more rewarding meal than an equivalently sized invertebrate, and especially energetically and nutritionally rewarding given the size of the meal particularly where fish are easily acquired. Fish may, thus, represent a ‘big ticket item’ in the nutritional budget of semi-aqautic spiders. Feeding on fish may be particularly advantageous during the mating period when the elevated energy and protein requirements of gravid female spiders require increased food intake [13] or at times of limited availability of invertebrate prey when feeding frequency is otherwise depressed and cannibalism elevated [100]–[101]. Complete piscivory is probably rare and restricted to those occasions when semi-aquatic spiders gain easy access to small fish kept at high density in artificial rearing ponds or aquaria [18], [131] or in small shallow waterbodies (see references above pertaining to *Ancylometes*). Additional research will be needed to reveal the extent and nutritional importance of fish in the diet of these spiders.

**Table 4:** Estimated fresh weight (g/prey item) and caloric value (kJ/g dry weight) of different prey categories used by semi-aquatic spiders.

Prey category	Fresh weight (g/prey item)	Caloric value (kJ/g dry weight)	Source
Freshwater fish (Teleostei)	<1–7	21–24	[177]
Other vertebrates (tadpoles, frogs)	<1–9	21–25	[178]–[179]
Crustaceans (crayfish, shrimps)	1–6	12–18	[177], [180]
Water striders (Gerridae)	∼0.03	N/A	[181]–[182]
Backswimmers (Notonectidae)	∼0.01–0.1	24	[183]–[185]
Water boatmen (Corixidae)	∼0.02–0.05	22	[181], [186]
Water beetles (Dytiscidae)	<0.01–0.02	22	[181]–[182]
Water scavenger beetles (Hydrophilidae)	N/A	23	[181]
Caddisflies (Trichoptera)	<0.01–0.04	22–23	[181]–[182], [184]
Mayflies (Ephemeroptera)	<0.01–0.06	22–23	[177], [181]–[182]
Stoneflies (Plecoptera)	<0.01–0.2	21–22	[177], [182]
Midges (Chironomidae)	≤0.01	21–23	[181]–[182], [184]
Mosquitoes (Culicidae)	<0.01	22	[181], [187]
Dragonflies, Damselflies (Odonata)	0.1–1.5	21–22	[177], [181], [184], [188]

### How Important are Semi-Aquatic Spiders in Aquatic Food Webs?

Riparian zones are widely recognised as ecotones of high productivity, diversity and ecological importance and cross boundary transfer of material from terrestrial and aquatic environments is frequently fundamental to the nature of the food webs of both [140]–[143]. Spiders do occur in the diet of fish specialising in the consumption of terrestrial invertebrates that inadvertently enter and subsidise food webs of the aquatic environment [58], [144]–[147] and semi-aquatic spiders serve as food for numerous aquatic and semi-aquatic vertebrate predators such as juvenile crocodilians [148]–[152], marshsnakes [153], anurans [145], wading birds such as herons [29], [154], and passerine birds [155]. Furthermore, semi-aquatic spiders may compete with aquatic predators for insect prey floating on the water surface (e.g., *Dolomedes* spp. versus sunfish *Lepomis* spp. [144], [156]). Apart from fish, a variety of aquatic crustaceans (i.e., crayfish, crabs, shrimps, and amphipods) are consumed by these spiders [19], [78], [157]–[159] and furthermore the adult stage of many aquatic insects is consumed by semi-aquatic spiders. Adults of aquatic insect species greatly enhance the overall abundance of insects in riparian zones [160] and this subsidy allows spider abundance, biomass and diversity to be significantly elevated (see Sanzone et al. [161]). Semi-aquatic spiders are an important component of freshwater and terrestrial food webs with multiple linkages within and between both [39], [100], [162].

## Concluding Remarks

It has been long-known that semi-aquatic spiders of the family Pisauridae occasionally predate small fish; however, past studies focused on just two genera of a single family (i.e., *Dolomedes* and *Nilus* [8], [163]–[164]). We found that the diversity of spider families engaged in fish predation is much higher than previously thought and encompasses at least eight spider families. Fish predation by spiders is geographically widespread but largely limited to the warmer areas between 40° S and 40° N. Semi-aquatic spiders capture a wide diversity of fish species but are constrained in the size of prey they can capture. The capture and consumption of fish by spiders represents a significant departure from the average dietary patterns and predator-prey size ratios reported in the literature and fish might be an occasional prey item of substantial nutritional importance. A better understanding of the nutritional ecology of the semi-aquatic spiders and their ecosystem role is needed.

## Acknowledgments

Many people have contributed to this review paper. First we wish to thank those scientists who identified fishes based on photographs - including James Albert (University of Louisiana), Jeanette Carpenter Haegele (USGS Denver Field Station), Glen Collier (University of Tulsa), Dean Hendrickson (University of Texas), Jeffrey Hill (University of Florida), Brett Johnson (Colorado State University),

Frank Jordan (Loyola University New Orleans), Mark Kennard (Griffith University), Maurice Kottelat (National University of Singapore), Edith Marsh-Matthews (University of Oklahoma), Larry Page (Florida Museum of Natural History), Lynne Parenti (National Museum of Natural History), Andrew Simons (University of Minnesota), Rainer Sonnenberg (Zoologisches Forschungsinstitut Alexander Koenig), Donald Stewart (State University of New York), Melanie Stiassny (American Museum of Natural History), and Richard Vari (National Museum of Natural History). Antonio Brescovit (Instituto Butantan), Ansie Dippenaar-Schoeman (University of Pretoria), G.B. Edwards (Florida State Collection of Arthropods), Hubert Höfer (Staatliches Museum für Naturkunde Karlsruhe), Kelly Kissane (Blinn College), Astri Leroy (The Spider Club of Southern Africa), Robert Raven (Queensland Museum), Adalberto Santos (Universidade Federal de Minas Gerais), and Estevam L. Cruz da Silva (Field Museum of Natural History) identified spider species in the photographs. Tadashi Miyashita (University of Tokyo) conducted a survey among spider researchers in Japan, using a mailing list of “kumo-net”. Appreciation is also expressed to the following people who helped us in various ways: David Dudgeon (University of Hong Kong), Thierry Gasnier (Universidade Federal do Amazonas), James Harwood (University of Kentucky), Alison King (Charles Darwin University), Mirjam Knörnschild (University of Ulm), Witold Lapinski (University of Ulm), Markus Metz (University of Ulm), Dolores Schuetz (University of Bern), Helen Smith (www.dolomedes.org.uk), I-Min To (Tunghai University), and Jakob Walter (Fischzuchtanstalt Neuhausen) and of course, the many people who provided unpublished information and photographs.

## Supporting Information

File S1 **Detailed Reports Description.**
(DOC)

Flow Diagram S1 **PRISMA 2009 Flow Diagram.**
(DOC)

Checklist S1 **PRISMA 2009 Checklist.**
(DOC)
