# Supplementary material for: Fish Predation by Semi-Aquatic Spiders: A Global Pattern
Source: PLoS One. 2014 Jun 18;9(6):e99459. doi: 10.1371/journal.pone.0099459 (PMC4062410; doi:10.1371/journal.pone.0099459)
Supplement: File S1 — Detailed Reports Description. (DOC) [file pone.0099459.s001.doc]

**SUPPORTING INFORMATION FILE S1**

**Fish Predation by Semi-Aquatic Spiders: A Global Pattern - Martin Nyffeler & Bradley J. Pusey**

**DETAILED REPORTS DESCRIPTION**

**NORTH AMERICA**

**# 1), # 2), # 3), # 4), # 5), # 6), # 7) and # 8) –** In the Steinhart Aquarium building in San Francisco, California, a total of eight pigmy sunfish (*Elassoma zonatum*) had been fished out of a balanced aquarium by a *Dolomedes* spider within just a few days (Adams 1927; Gudger 1931). The spider was seen feeding on these fish.

**# 9) –** Andreas Kettenburg (Thousand Oaks, California) photographed a fishing spider consuming a small fish in a small stream in the Pajarito Mountains of Arizona in Sycamore Canyon, at the US / Mexican border (Fig. 3A). The spider was photographed in a very shallow side section of the stream within a fairly steep canyon with a good tree cover in that particular area (photo taken between 12:00-03:00 PM). The photographer did not even notice that the spider was feeding on a fish until he post processed the image after his return from that trip. The spider in question is most likely *Dolomedes triton*, identified by Kelly Kissane and Mandy Howe. Based on the principle of exclusion of the fishes occurring in this river, the fish shown in the photo is most likely a Sonora chub (*Gila ditaenia*) identified by Jeanette Carpenter Haegele and Dean Hendrickson.

**# 10) –** In the Life Pond Aquarium located at the Brazos Bend State Park in Texas a *Dolomedes triton* was photographed in the act of devouring a golden topminnow (*Fundulus chrysotus*) (Richard Dashnau, pers. comm.). The aquarium has a content of a little over 100 Liter. The spider identification was verified by Kelly Kissane. The fish was identified by Richard Dashnau as *Fundulus chrysotus*; this identification has been confirmed by Jeffrey Hill and Larry Page. The fish was ~1.2 times as long as the spider and might have measured ~2.5-3 cm in length.

**# 11) –** On January 4, 2008, Leslie Todd photographed an incidence whereupon a *Dolomedes triton* was eating a goldfish on the edge of the fish pond in her garden in Greenville, Texas. The fish had an estimated total length of ~5 cm. The photo has been posted on Mrs. Todd’s flickr website. The spider has been identified by Kelly Kissane.

**# 12) –** In one of the channel catfish (*Ictalurus punctatus*) rearing ponds of the fish hatchery in Tishomingo, Oklahoma, many young fish were captured and eaten by *Dolomedes triton* spiders (Meehean 1934). Apparently the spiders caused considerable damage, for all spiders living in and near the rearing pond had to be eradicated.

**# 13) –** Meehean (1934) reports that a *Dolomedes* sp. had captured a small fish (*Gambusia patruelis* = *Gambusia affinis*). This observation was made in a thick growth of duckweed in a bayou below Chaplin’s Lake at Natchitoches, Louisiana, USA.

**# 14) –** Meehean (1934) reports that he found a *Dolomedes* sp. with a small sunfish prey *Lepomis macrochirus* (formerly *Helioperca incisor*) in a fish pond at the Hatchery at Natchitoches, Louisiana, USA.

**# 15) –** Suhr & Davis (1974) report an incidence of fish predation by a *Dolomedes triton* witnessed in Mississippi, USA. The captured fish was a mosquitofish (*Gambusia affinis*).

**# 16) –** Gudger (1922) reports an incidence where a *Dolomedes* sp. was capturing a minnow. This was witnessed at the edge of a freshwater pool filled by water from a spring near Courtland, Lawrence County, Alabama, USA.

**# 17) –** Paul Moler (Florida Fish & Wildlife Conservation Commission, Gainesville) and his colleague Barry Mansell took a photo of a *Dolomedes* sp. (*Dolomedes triton*?) with a mosquitofish prey (*Gambusia affinis*) in Washington County, Florida, USA (30.79° N, 85.76° W). They were sampling the pond water and vegetation with a wire-bottomed box seine. When they removed the device from the water, several small fish were flopping around on the wire and a *Dolomedes* spider was trapped as well; immediately after that the spider grabbed one of the fish (Paul Mohler, pers. comm.). This freshwater pond was originally dominated by cypress, but the trees had been previously removed. Spider and fish were collected along the margin of the pond where the vegetation consisted of emergent grasses.

**# 18) –** During extensive sampling of floating vegetation for aquatic salamanders throughout North Florida, USA, still other incidences of fish predation by *Dolomedes* sp. were witnessed (Paul Mohler, pers. comm.).

**# 19) –** In 2008, Claire Sunquist-Blunden (Ocala, Florida) took a photo of a *Dolomedes triton* with a little fish as prey (Fig. 2C). She found this spider at a rural boat ramp in Citrus County, Florida, USA. The boat ramp was in a neighbourhood canal in the Tsala Apopka Lake (Chain of Lakes). Mrs. Sunquist-Blunden observed the spider at the edge of the canal in littoral vegetation and basically flushed it out into the open habitat at the edge of the ramp. The spider identification was verified by Kelly Kissane. The fish was identified by Jeffrey Hill, Frank Jordan, Edith Marsh-Matthews, and Larry Page to be probably a least killifish (*Heterandria formosa*).

**# 20) –** On May 26, 2006, Machele White took two photos of a female *Dolomedes triton* with a small fish prey in a garden pond in Lady Lake, Lake County, Florida, USA. The photos (Figs. 2B, D) were taken while Mrs. White was cleaning out some of the Water lettuce. The spider identification was verified by Kelly Kissane. Based on the photos the fish was identified by Jeffrey Hill, Brett Johnson, Frank Jordan, Edith Marsh-Matthews, Larry Page, Andrew Simons, and Donald Stewart to be probably a mosquitofish (*Gambusia holbrooki*). The fish was ~2 times as long as the spider.

**# 21-23) –** Barbour (1921) witnessed three incidences of fish predation by *Dolomedes* spiders in a shallow swampy area in the upper St. John River, above Lake Washington, Florida, USA. All three incidences were witnessed in just one afternoon. The fish killed by the spiders were mosquitofish (*Gambusia*) of about the same length as the spider. The spider was identified by Barbour (1921) as *Dolomedes tenebrosus*. Based on location, however, Carico (1973) stated that the spider species in question was most likely *Dolomedes okefinokensis*.

**# 24) –** In late August 2006, Stacy Cyrus took a photo of a *Dolomedes triton* with a mosquitofish (*Gambusia* sp.) caught in a little backyard pond in Tampa, Florida, USA (photo posted on the website of ‘Davesgarden.com BugFiles’). After catching the fish, the spider dragged it up onto a lily pad where it was devoured (Fig. 2A).

**# 25)** **–** Wildlife photographer Brian Kenney, North Port, Florida, observed *Dolomedes triton* feeding on fish in an inland freshwater swamp (Highlands Hammock State Park, Florida, USA) sometime between 1995 and 2000. On this location, 1 or 2 incidences of fish predation have been witnessed. The spiders were using tree trunks in standing water as a fishing platform. The spiders held on to the tree trunk with their hind legs, but rested on the surface of the water with their front legs.

**# 26) –** Brian Kenney (pers. comm.) observed *Dolomedes triton* feeding on fish on an inland freshwater pond (near Bradenton, Florida, USA) sometime between 1995 and 2000. On this location, 3 or 4 incidences of fish predation have been witnessed. The spiders were using cattails (*Typha domingensis*) as fishing sites. Bryan Kenney stated “The spider moved to the lower end of the cattail, even with the water’s surface, anchored its body with its hind legs and spread out on the surface of the water, appearing to either feel for vibrations in the surface of the water or create them to attract prey. When a fish came close, it moved slightly higher on the cattail and jumped on it, then dragged the fish up out of the water higher on the cattail to eat it. The sequence was the same for the spiders fishing from tree trunks” (see report # 25). The aquatic vegetation in the area was primarily water hyacinth, water lettuce, and an aquatic fern similar to duckweed.

**# 27) –** Brian Kenney (pers. comm.) observed *Dolomedes triton* feeding on fish on the Myakka River (near Venice, Florida, USA) sometime between 1995 and 2000. On this location, 2 or 3 incidences of fish predation have been witnessed (Brian Kenney, pers. comm.). The spiders were using cattails (*Typha domingensis*) as fishing sites.

**# 28) –** On April 27, 2008, Misti and Chris Little (Stagecoach, Texas) found a *Dolomedes* sitting on a tree that was devouring a small fish. This incidence was witnessed and photographed in a swamp at Big Cypress National Preserve in South Florida, USA (Fig. 2F; Misti Little, pers. comm.). The spider was identified by Kelly Kissane as *Dolomedes okefinokensis*. Based on photos the fish was identified by Jeffrey Hill, Frank Jordan, Edith Marsh-Matthews, Larry Page, and Andrew Simons to be probably a mosquitofish (*Gambusia holbrooki*). The fish was ~1.8 times as long as the spider and may have measured ~4-4.5 cm in length. Mist Little stated “We have seen them predating on various fishes or crustaceans near where the water meets the trees during the summertime. Typically the waters recede in the ‘dry’ months of the year, late winter through very early summer in a normal year and the spiders are still around and not confined to the trees”. Chris Little took a photo of a specimen of *Dolomedes* feeding on a crayfish.

**# 29) –** Gudger (1931) reports an incidence witnessed near Atlanta, Georgia, USA. A *Dolomedes* sp. was seen capturing a small minnow in a small pool.

**# 30) –** On May 20, 2011, Patrick Randall (Fort Bragg, North Carolina) photographed a *Dolomedes triton* preying on a small fish (Fig. 2E). This incidence was observed on the edge of a small, slow-moving stream in Fort Bragg (near Fayetteville, North Carolina, USA) that becomes almost dry during the hotter months of summer and fills up after it rains in cooler months. The area around the stream was covered with low vegetation; on one side was a dirt trail 10 m from the water’s edge, and on the other side was pine forest about 50 m away (Patrick Randall, pers. comm.). The spider had already captured the fish when this incidence was discovered a short time before midnight. The spider identification was verified by Kelly Kissane. Based on the photo the fish was identified by Jeffrey Hill, Frank Jordan, Edith Marsh-Matthews, Larry Page, and Andrew Simons to be probably a mosquitofish (*Gambusia holbrooki*). The fish was ~2.3 times as long as the spider and might have measured ~4.5-5.5 cm in length.

**# 31) –** In the Trout Hatchery of the U.S. Bureau of Fisheries at Erwin, Tennessee, USA, it was witnessed that a *Dolomedes* sp. had killed a trout fingerling (*Salmo irideus* or *Salmo shasta*) in a small trout pond (Gudger 1925). The fish had a length of ~5.7 cm, whereas the spider’s body (without legs) measured ~1.9 cm in length.

**# 32) –** There is annectodal evidence that trout fry (*Salmo irideus* or *Salmo shasta*) were eaten by *Dolomedes* sp. in the Hatchery of the U.S. Bureau of Fisheries at Erwin, Tennessee, USA (Gudger 1925)

**# 33) –** On June 28, 2012, Jason Butler (Lexington, KY) observed and photographed a *Dolomedes tenebrosus* eating a creek chub (*Semotilus atromaculatus*) on the bank of Bullskin Creek near Brutus, Kentucky, USA (Fig. 3B). The spider was identified by Kelly Kissane to be a female *Dolomedes tenebrosus*. The fish was ~3 times longer than the spider and might have measured ~6-7.5 cm in length. The fish was identified by Jason Butler and his identification was later confirmed by several ichthyologists specialized in *Semotilus* biology.

**# 34) –** In spring 2012, Jason Butler (Lexington, KY) observed a *Dolomedes* sp. eating a mosquitofish (*Gambusia affinis*) at Lick Creek in Henderson County, Kentucky, USA. The fish was identified by Jason Butler (pers. comm.).

**# 35) –** Krupa & Sih (1998) reported to have seen *Dolomedes* sp. capture mosquitofish *Gambusia affinis* in Kentucky.

**# 36) –** Gudger (1922) reports an incidence whereupon a semi-aquatic spider (*Dolomedes* sp.) was observed capturing fish in a dyke situated in a swampy wood on the South Amboy shore in New Jersey, USA. The spider had a body length of 1.9 cm (0.9 g live weight) and the fish of 8.3 cm (4.3 g live weight).

**# 37) and # 38) –** In a building in Philadelphia, Pennsylvania, USA, two sunfish (Centrarchidae), each ~5 cm in length, were kept in a basin (Gudger 1922). One day it was witnessed how a semi-aquatic spider (*Dolomedes* sp.) entered the basin and started attacking the two fish. [Some North American *Dolomedes* are indeed known to occasionally intrude into the moist basements of houses (Carico 1973).] The spider first attacked the first and immediately thereafter the second fish. Though the spider was chased away, the two fish died within a few hours after their encounter with the spider (Gudger 1922).

**# 39) –** On May 10, 1890, a semi-aquatic spider (*Dolomedes* sp.) present on the water surface of a small, wood-shaded pond on Staten Island, New York, USA, was observed catching a small fish, ~2.5 cm in length (Davis 1891; Gudger 1922). This incident took place in the late afternoon.

**# 40) –** On August 28, 2010, Jeffrey Hollis (East Haddam, Connecticut , USA) was with his family visiting a house in Turtle Cove on Sebago Lake in Maine, USA (43.89° N, 70.50° W) when he witnessed the following (Fig. 3G): “The family did a fair bit of fishing off the dock that weekend, and a mis-cast resulted in the bait fish landing just off the edge of the dock, and when they pulled it up to attempt another cast, the spider scuttled out very quickly from underneath the dock to attack the fish. This took place at 09:25 AM. It was over quickly, though, and the spider went back under the dock about a second or two afterwards.” Based on a photo the fish was identified by Jeffrey Hill, Brett Johnson, Larry Page, Andrew Simons, and Donald Stewart to be probably a golden shiner (*Notemigonus crysoleucas*), a common bait fish used in North America. The spider was identified by Kelly Kissane as being probably *Dolomedes tenebrosus*.

**# 41) –** Mohrhardt (1963) published a photo depicting a specimen of *Dolomedes scriptus* preying on a minnow along a Michigan stream.

**# 42) –** Tod Lewis (Austin, Texas) observed the following: He was standing on the dock on the shoreline of Flambeau river near Ladysmith, Wisconsin, USA, when he heard a splash in the water. He looked at the source of the ripples and saw a spider dragging a fish out of the water on to a log (Figs. 3C-D; Tod Lewis, pers. comm.). This incident took place between 03:00-05:00 PM. The surrounding environment is an old forest with a mix of pine, elm, maple and other trees. The river is dammed up with a hydro-electric plant on the lower side. Based on photos the fish was identified by Jeffrey Hill and Larry Page to be a smallmouth bass (*Micropterus dolomieu*). On this location, smallmouth bass are the fish most frequently caught by fishermen (Tod Lewis, pers. comm.). The spider in the photo is possibly *Dolomedes scriptus*, identified by Kelly Kissane. The fish was ~2 times as long as the spider and might have measured ~4-5 cm in length.

**# 43) –** National Geographic photographer Harry Ellis took a picture of a specimen of *Dolomedes vitattus* devouring a fish on the edge of a shallow water body on a location not otherwise specified in the eastern part of USA. The photo had been included in a paper by Zahl (1971). The spider was identified by Kelly Kissane to be a male *Dolomedes vitattus*. The fish was ~2.5 times longer as the spider and might have measured ~5-6 cm in length.

**# 44) –** Carico (1973) had witnessed a female *Dolomedes scriptus* feeding on a small darter (Percidae). He does not provide any information on the location or habitat in USA where this incidence had been witnessed.

**# 45) –** An anonymous blogger photographed a *Dolomedes scriptus* with fish prey resting on a pole. The photo was taken somewhere in North America on a location not otherwise specified. Available: <http://www.flickr.com/photos/44608110@N06/4114197379/>. Accessed 4 April 2013. The spider was identified by Kelly Kissane as *Dolomedes scriptus*. The fish could not be identified to family but several ichthyologists, to whom the photo was shown, stated that it is a species belonging to the infraclass Teleostei.

**# 46) –** Lloyd Alter (Toronto, Canada) took several photos of a *Dolomedes scriptus* feeding on a fish (Figs. 3E-F) on July 1, 2008 (at 10:40 AM), at the end of a 4.8 m long dock located at Shoe Lake near Dorset, Lake of Bays, Ontario, Canada (Lloyd Alter, pers. comm.). Shoe Lake is a freshwater lake and the water at the end of the dock is about 1.8 m deep (Fig. 3E). It was relatively calm that day but not flat water. Based on photos the fish was identified by Jeffrey Hill, Brett Johnson, Frank Jordan, Larry Page, Andrew Simons, and Donald Steward to be probably a green sunfish *Lepomis cyanellus* (Centrarchidae). The spider was identified by Kelly Kissane as *Dolomedes scriptus*. The fish was ~2.5 times as long as the spider and might have measured ~5-6 cm in length.

**CENTRAL AND SOUTH AMERICA (NEOTROPICS)**

**# 47) –** A specimen of *Ancylometes bogotensis* has been witnessed and photographed feeding on a poeciliid fish at the edge of a stream flowing through tropical forest near Tirimbina, Costa Rica (Lapinski & Tschapka, 2013; Witold Lapinski, pers. comm.).

**48) –** On August 26, 2011, Jessica Stapley (University of Sheffield, UK) witnessed and photographed a spider resting on a tree trunk devouring a fish (Fig. 5C). This was on the edge of the Rio Frijoles river on Pipeline road, Central Panama, surrounded by lowland tropical rainforest (Jessica Stapley, pers. comm.). The spider was identified by Antonio Brescovit, Hubert Höfer, Adalberto Santos, and Estevam L. Cruz da Silva to be a *Trechalea* sp. The fish was identified by Melanie Stiassny and Richard Vari to be a species in the order Characiformes. The fish was ~2.2 times as long as the spider.

**# 49) –** Gudger (1925) reports an observation from Panama whereupon it was witnessed how a semi-aquatic spider has been observed capturing a fish of the order Characiformes. In our opinion the spider in question fits the description of Trechaleidae.

**# 50) –** On July 2, 2009, Juan Esteban Arias A. (Cali, Colombia), during the daylight hours, witnessed and photographed a spider capturing a small fish in a shallow, small stream (nearly dried out by the summer heat) near Quebrada Valencia, Magdalena, Colombia (Fig. 5B). The spider was identified by Antonio Brescovit, Hubert Höfer, Adalberto Santos, and Estevam L. Cruz da Silva to be a *Trechalea* sp. Melanie Stiassny and Richard Vari identified the fish as a species in the order Characiformes.

**# 51) –** On September 10, 2011, Solimary Garcia Hernandez (Instituto de Biociências, Universidade de São Paulo, Brazil) witnessed and photographed a spider preying on a fish (Figs. 5A, E). This occurred at approx. 04:00 PM (Solimary Garcia Hernandez, pers. comm.). The spider was standing on a rock in the middle of a small river near Paratebueno, state of Cundinamarca, Colombia (Fig. 5E). The spider was identified by Antonio Brescovit, Hubert Höfer, Adalberto Santos, and Estevam L. Cruz da Silva to be a *Trechalea* sp. Melanie Stiassny and Richard Vari identified the fish as a species in the order Characiformes. The fish was ~2.2 times as long as the spider.

**# 52) –** September 23, 2007, at 08:19 PM, Ed Germain (Sydney, Australia) witnessed and photographed an incidence of fish predation by a spider near the Samona Lodge, Cuyabeno Wildlife Reserve, Ecuador, during a night-walk (completely dark) through medium density rainforest that would have been difficult to penetrate without a path (Fig. 4C). However, there were clear spaces of leaf litter and networks of puddles on the forest floor. The spider was encountered approximately 50-100 m from Samona Lodge, to the side of the path, at the very edge of a large puddle of clear water several meters in diameter, no deeper than 30 cm, with small plants breaking the surface (Ed Germain, pers. comm.). The spider was found because of its reflective eyes. From memory, the legspan was ~10-12 cm. It was right at the water’s edge, with the front legs in the water, and the fish already landed, under its body. The fish was still alive, giving feeble flicks with its tail. On being exposed to the light the spider moved away quickly, dragging the prey between its legs, moving over the vegetation. The spider was identified by Antonio Brescovit, Hubert Höfer, Adalberto Santos, and Estevam L. Cruz da Silva to be an *Ancylometes* sp. (Ctenidae). The fish was identified by Melanie Stiassny and Richard Vari to be a species in the order Characiformes. According to Richard Vari this fish belongs to the genus *Cyphocharax* (Curimatidae). The fish was ~2.4 times as long as the spider and may have measured ~6 cm in length. Two days earlier, at 08:19 PM, a specimen of apparently the same spider species had been seen and photographed predating a large tadpole at the same location.

**# 53) –** In early January 2011, during a night walk through the jungle Tim Wohlberg (Kelowna, British Columbia, Canada <http://zufalladventures.com/>) witnessed and photographed an incidence of fish predation by a spider a few km away from the Samona Lodge, Cuyabeno Wildlife Reserve, Ecuador. The photo (Fig. 4D) was taken about 2 meters from a small creek. In the light of his headlamp he saw something moving slowly, not as fast as a spider would normally move, and when he went closer he saw the half eaten fish (Tim Wohlberg, pers. comm.). Based on photos the spider was identified by Antonio Brescovit, Hubert Höfer, Adalberto Santos, and Estevam L. Cruz da Silva to be an *Ancylometes* sp. (Ctenidae). The fish was identified by Melanie Stiassny and Richard Vari to be a species in the order Characiformes.

**# 54) –** On June 7, 2011, Cleatus Cobb witnessed and photographed an *Ancylometes* sp. preying on a fish in the Cuyabeno Wildlife Reserve, Ecuador. Available: <http://www.flickr.com/photos/cleatus_cobb/6837886624/>. Accessed 15 June 2013. The fish was identified by Richard Vari as belonging with high likelihood to the order Characiformes. The spider was identified by Antonio Brescovit and Hubert Höfer to be an *Ancylometes* sp. (Ctenidae).

**# 55)** **–** On September 11, 2010, an anonymous blogger observed and photographed on September 11, 2010, during a night treck, an *Ancylometes* sp. eating a fish not far away from Samona Lodge, Cuyabeno Wildlife Reserve, Ecuador. Available: <http://alexlisasouthamerica.blogspot.ch/2010/09/amazon-jungle-adventure.html>. Accessed 15 June 2013. The fish has been identified by Richard Vari as belonging to the order Chariciformes. The spider was identified by Antonio Brescovit and Hubert Höfer to be an *Ancylometes* sp. (Ctenidae).

**# 56) –** In October 2012, an anonymous blogger, travelling in Cuyabeno, Ecuador, saw during the night a large, semi-aquatic spider capture and eat a fish. Based on the location and circumstances the spider in question was with high likelihood an *Ancylometes* sp. Available: <http://www.tripadvisor.com.au/ShowUserReviews-g303844-d3438372-r148106445-Guacamayo_Ecolodge-Cuyabeno_Sucumbios_Province.html> Accessed 4 November 2013.

**# 57) –** On May 23, 2011, at about 08:00 PM, Craig Harrison went for a night hike in the Cuyabeno Wildlife Reserve, Ecuador. In marshy area approximately 500 meters away from Samona Lodge, he photographed a ctenid spider with a fish prey nearly twice the spider’s size (Fig. 4A). Available: <http://www.offexploring.com/funandgamesaroundtheworld/blog/ecuador/tipishca/2011-05-23+21%3A14%3A30>. Accessed 15 June 2013, and <http://media.offexploring.co.uk/photos/funandgamesaroundtheworld/photos/040611-t> . Accessed 15 June 2013. The fish was identified by Richard Vari as a catfish most likely in the family Pimelodidae (Siluriformes). The spider was identified by Antonio Brescovit, Hubert Höfer, and Estevam L. Cruz da Silva to be an *Ancylometes* sp. (probably *A. rufus*).

**# 58)** **–** In central Amazonia (Reserva Ducke, Manaus), Brazil, an incidence of an *Ancylometes rufus* preying on a fish was witnessed (Höfer & Brescovit 2000; Hubert Höfer, pers. comm.).

**# 59) –** Around 1980, Jacques Jangoux (Belém Area, Brazil) took a photo of a *Trechalea* sp. eating a small fish on the bank of the Rio Maicuru river, tributary of the Amazon coming down from the Guiana Highlands in Para State, Brazil (Fig. 5D). This incidence was observed in the midst of tropical rainforest where slowly running water with pools of standing water between rocks occurred (Jacques Jangoux, pers. comm.). The spider was identified by Antonio Brescovit, Hubert Höfer, Adalberto Santos, and Estevam L. Cruz da Silva to be a *Trechalea* sp. Melanie Stiassny and Richard Vari identified the fish as a species in the order Characiformes. The fish was ~1.8 times as long as the spider.

**# 60) –** Alfredo Dosantos Santillan (c/o Amazonia Expeditions, Tampa, Florida) observed and photographed a ctenid spider that had captured a fish in the Tahuayo river area de Conservacion Regional Communal Tamshiyacu-Tahuayo Loreto, Peru (Paul Beaver, pers. comm.; Fig. 4B). Available: <http://thinkjungle.com/amazon-rainforest-life/amazon-rainforest-carnivores/>. Accessed 24 September 2013. The spider was identified by Antonio Brescovit and Hubert Höfer to be most likely an *Ancylometes* sp. Richard Vari identified the fish as a species in the order Characiformes. The fish is ~2.7 times as long as the spider.

**AUSTRALIA**

**# 61) and # 62) –** In February 1935, it was witnessed that a semi-aquatic spider (*Dolomedes* sp.) had captured a red goldfish of ~7 cm length in a fish pond in Adelaide, Australia (McKeown 1943). Only one week later, a second goldfish, this time ~7.5 cm in length, had been captured by the same type of spider.

**# 63) –** McKeown (1943) witnessed that *Dolomedes facetus* resting on the water surface of calm pools on the Hacking River near Audley, New South Wales, Australia, was sometimes catching small fish.

**# 64) –** In 1932, it was reported to the Australian Museum that a goldfish had been killed by a *Dolomedes facetus* in North Sydney, Australia. The fish measured ~9 cm in length and was strongly mutilated behind the head (McKeown 1943).

**# 65) –** In 1941, the Australian Museum was informed that a fancy type goldfish, ~2.5 cm in length, had been captured by a semi-aquatic spider (*Dolomedes* sp.) in a goldfish pool in a garden in Lismore, New South Wales (McKeown 1943). The spider was devouring the fish while resting on a lily leaf.

**# 66) –** Loren Jarvis (near Brisbane, SE Queensland) photographed a *Dolomedes* sp. preying on a Mountain Galaxias (*Galaxias olidus*) (Fig. 6B). The location description is as follows: North Branch Creek, Goomburra, Main Range National Park, SE Queensland, Australia (Loren Jarvis, pers. comm.). This location is in subtropical rainforest. The spider was photographed on the bank of the creek in an area where water movement was minimal. Based on a photo the fish was identified by Mark Kennard and Bradley Pusey, the spider by Robert Raven. The fish was ~3.4 times as long as the spider and might have measured ~7-8.5 cm in length.

**# 67) –** In a garden fish pond in Moffat Beach near Brisbane, Queensland, Australia, Peter Liley (pers. comm.) observed the following: On December 13, 2008, a fishing spider was feeding on a pond fish (genus *Xiphophorus*) that it had pulled onto a lilypad (Fig. 6A). The edge of the pond is about 45 cm above the surrounding garden. *Lomandra* plants are growing along the eastern side. The spider was seen waiting for prey with 4 forelegs on the surface of the water and 4 backlegs holding onto the lilypad and some other foliage. The fish was identified by James Albert based on a photo. The spider was identified by Robert Raven to be *Dolomedes facetus*. The fish was ~2.2 times as long as the spider and might have measured ~5-6 cm in length.

**# 68) and # 69) –** Bradley Pusey (University of Western Australia) witnessed pisaurid spiders catch rainbowfish juveniles (*Melanotaenia* spp.) and blueeyes (*Pseudomugil* spp.). The rivers in which these observations were made were the Mulgrave and Nth Johnstone Rivers between Innisfail and Cairns, Queensland, Australia (Bradley Pusey, pers. comm.).

**# 70)** **–** Photographer Jean-Paul Ferrero took photos of a *Dolomedes facetus* with fish prey on a location in Australia not otherwise specified (posted on the website of ardeaprints.com). The spider has been identified by Robert Raven based on a photo.

**# 71) –** There is an annectodal report from the first half of the 19th century whereupon large semi-aquatic spiders were seen catching fish in Australian forest swamps (cited by McKeown 1943). This report appears to be credible if one takes into account that the phenomenon of fish predation by spiders was unknown to science in the first half of the 19th century and that this was later fully confirmed for Australia (see reports # 61-70).

**ASIA**

**# 72) –** In 2006, photographer Michael Lo (City of Kuching, Malaysia) was exploring the eastern Batang Sadong basin in Borneo, when he found a pisaurid spider eating a juvenile *Rasbora* fish while sitting on a horizontal leaf on the edge of a shallow forest river (Fig. 6C). The fish was ~3.5 times as long as the spider. According to Maurice Kottelat the fish in question is apparently *Rasbora calliura*.

**# 73) –** A record of fish predation by *Dolomedes pallitarsis* was published by Hisao Akabane (1995). On May 23, 1993, he found a female *Dolomedes pallitarsis* feeding on a fish (*Pseudorasbora parva*; Cyprinidae) in Atsugi city [near Tokyo], Kanagawa prefecture, Japan. The fish had a body length of 3.3 cm. The spider was found on the leaf of an emergent plant about 20 cm above the water surface. Meanwhile, the name *Dolomedes pallitarsis* has been changed to *Dolomedes saganus* (Platnick 2013).

**# 74) –** I-Min Tso (Tunghai University, Taichung, Taiwan) communicated to us that his research group had observed a female *Dolomedes raptor* catch and eat a fish on the edge of a stream winding through low elevation subtropical forest near Tung-Shih, Taichung county, Taiwan (I-Min Tso, pers. comm.). This incidence has been photographed by graduate student Tai-Shen Lin (Fig. 6D). The fish in question was a teleost fish ~2.2 times as long as the spider.

**# 75) –** Dudgeon (1999) mentions *Dolomedes pallitarsis* as a predator of the rice fish (*Oryzias curvinotus*) in the Hong Kong area.The name *Dolomedes pallitarsis* has been changed to *Dolomedes saganus* (Platnick 2013).

**# 76) –** David Dudgeon (Hong Kong University) told us that he has seen a pisaurid spider capturing and eating a fish, specifically a small individual of the stream minnow *Parazacco spilurus* (Cyprinidae), at the water’s edge. The fish was ~2.5 cm in length. The observation was made in Hong Kong along the margins of a shallow, stony stream (actual location was Pak Tam Chung Stream) (David Dudgeon, pers. comm.).

**# 77) –** Jiang et al. (2013) reported that a semi-aquatic spider (*Dolomedes mizhoanus*) was seen capturing a fish in the Guangxi province, China. This spider species was also seen feeding on fish in captivity.

**# 78) –** In India, Bhattacharjee (1931-32) reported a case where a small *Elassoma* fish was captured by a specimen of the lycosid spider *Pardosa pseudoannulata*.

**AFRICA**

**# 79) –** In South Africa, Astri Leroy (pers. comm.) has seen a *Nilus curtus* with fish prey. She stated: “At Rooikoppies Dam in Northwest Province, South Africa, during the summer when the dam is really full, the shallows are about knee height and extend for many meters from the shore. Sedges and grasses poke above the water surface and there are many fishing spiders, I suspect *Nilus curtus*, resting, head-down on these plants with the front legs on the water surface. If one wades towards deeper water thousands of small fish are disturbed and the spiders dive in and catch them as they swim past. One really large female caught and subdued a fish that was probably over 4 cm long which must have been very much heavier than she was”. A photo of a *Nilus* sp. eating a fish in captivity is depicted in Leroy & Leroy (2003).

**# 80) –** Pickard-Cambridge (1903) reports that *Nilus* sp. was observed devouring trout fry in South Africa.

**# 81) –** Marcelo de Freitas (Cresta, South Africa) witnessed and photographed the following account: A semi-aquatic pisaurid spider, dangling from a lily flower bud, has pulled an unidentified fish (~4 cm in length) out of the water of a pond in the gardens of the Victoria Falls Hotel, Zimbabwe. This incidence was photographed in the evening, between 07:00-09:00 PM (Fig. 6E). The spider was identified as *Nilus massajae* by Ansie Dippenaar. The fish was ~1.7 times as long as the spider.

**# 82) –** On December 16, 2005, Duncan Reid (Yale University, USA) witnessed and photographed an attack by a semi-aquatic pisaurid spider on a killifish (Fig. 6F). The photo was taken in the southern part of Cameroon near the city of Kribi (Duncan Reid, pers. comm.) The habitat is a stream flowing through tropical secondary growth forest. Numerous semi-aquatic spiders were standing on the water surface. While Duncan Reid and his colleague Cyrille Dening were collecting fish with nets, many spiders got trapped in the nets as well. One of these spiders grabbed a killifish (Fig. 6F). The spider was identified as *Nilus* sp. by Ansie Dippenaar and Astri Leroy. The fish was identified by Glen Collier and Lynne Parenti as belonging to the genus *Aphyosemion*. The fish was ~2.2 times as long as the spider.

**# 83) and # 84) –** Romand (1980) reported that semi-aquatic pisaurid spiders had repeatedly been witnessed capturing specimens of the killifish *Aphyosemion walker* in a shallow streamlet flowing through secondary forest near Agboville, Ivory Coast. Romand took a photo of one such incidence. At the place, where the spider had captured the fish, the water was only 1-2 cm deep. The fish in the photo was ~1.5 times as long as the spider. Based on this photo the spider was identified as *Nilus* sp. by Ansie Dippenaar and Astri Leroy.

**EUROPE**

**# 85) –** Wildlife photographer Emanuele Biggi (Genova, Italy) found a *Dolomedes plantarius* feeding on a dead mosquitofish in Italy. He witnessed this in a dead branch of Po river in the Alessandria province, Northern Italy (Emanuele Biggi, pers. comm.). The spider already held a dead mosquito fish (*Gambusia*) probably taken in shallow water. The habitat was a large water body, with thick riverine vegetation (mostly *Phragmites*, *Typha*, and *Salix*) and some aquatic plants (*Salvinia* and *Nuphar luteus*). The water was densely populated by fish, most of them being mosquitofish.

**# 86) –** Berland & Pellegrin (1934) reported that a specimen of *Agroeca lusatica* (Liocranidae) was observed feeding on a trout fry in the fish breeding institution in Grattereau near Paris, France. This incidence must be considered rather unexpected, given that *Agroeca lusatica* is a species with preference for dry conditions (Duffey 2005).

**# 87) and # 88) –** Two incidences of fish predation by spiders were witnessed by Helen Smith (South Lopham, Norfolk, UK) while conducting an extensive conservation biology project (Smith 2000). The first incidence occurred in 2006; thereby Helen Smith (pers. comm.) witnessed and photographed that a gravid adult female *Dolomedes plantarius* had captured a stickleback (Fig. 7). This incidence occurred in a turf pond at Redgrave and Lopham Fen National Nature Reserve in East Anglia, UK. The second incidence occurred in 2011, at which time it was witnessed how an immature specimen of *Dolomedes plantarius*, only ~1 cm in length, was eating a small stickleback likewise at the Redgrave and Lopham Fen National Nature Reserve. The fish in question were *Pungitius laevis* identified by Maurice Kottelat.

**ADDENDUM**

**# 89) –** In around 1999-2002, Alison King (currently at Charles Darwin University) witnessed an incidence of fish predation by a pisaurid spider. She was sitting on the streambank of the Broken River - a tributary of the Murray River in Victoria near the town of Shepparton (36° 22’ 50’’ S, 145° 24’ 21’’ E), New South Wales, Australia - watching a small backwater, when she witnessed a group of about 5 pisaurids seemingly hunting in unison a school of small common carp (*Cyprinus carpio*) larvae into the shallows where one of them grabbed a larva. Only one spider was noticed feeding afterwards and the others just moved on. The fish prey was ~2 cm long and the spider had a body length of ~2 cm (cephalothorax plus abdomen) (Alison King, pers. comm.).

**REFERENCES**

Adams W (1927) Note on a fishing spider. Bull Zool Soc NY 30: 77.

Akabane H (1995) A record of predation of fish by *Dolomedes pallitarsis*. Kishidaia 69: 40-41 (in Japanese).

Barbour T (1921) Spiders feeding on small cyprinodonts. Psyche 28: 131–132.

Berland L, Pellegrin J (1934) Sur une araignée pêcheuse de poissons. B Soc Zool Fr 59: 210-212.

Bhattacharjee GC (1931-32) The fish-eating spiders in Bengal and their habits. Transact Bose Res Inst Calcutta 7: 238-249.

Carico JE (1973) The Nearctic species of the genus *Dolomedes* (Araneae: Pisauridae). Bull Mus Comp Zool 144: 435-488.

Carico JE (1993) Revision of the genus *Trechalea* Thorell (Araneae, Trechaleidae) with a review of the taxonomy of the Trechaleidae and Pisauridae of the Western Hemisphere. J Arachnol 3: 226-257.

Carico JE, Adis J, Penny ND (1985) A new species of *Trechalea* (Pisauridae: Araneae) from Central Amazonian inundation forests and notes on its natural history and ecology. Bull Brit Arachnol Soc 6: 289-294.

Davis WT (1891) A spider fisherman. Entomol News 2: 77.

Dudgeon D (1999) Tropical Asian streams: zoobenthos, ecology and conservation. Hong Kong: Hong Kong University Press. 844 p.

Duffey E (2005) Regional variation of habitat tolerance by some European spiders (Araneae) – a review. Arachnol Mitt 29: 25-34.

Gudger WE (1922) Spiders as fishermen. J Am Mus Nat Hist 22: 565-568.

Gudger WE (1925) Spiders as fishermen and hunters. J Am Mus Nat Hist 25: 261-275.

Gudger WE (1931) Some more spider fishermen. Nat Hist 31: 58-61.

Höfer H, Brescovit AD (2000) A revision of the neotropical spider genus *Ancylometes* Bertkau (Araneae: Pisauridae). Insect Syst Evol 31: 323-360.

Jiang L, Liu C, Duan Z, Deng M, Tang X et al. (2013) Transcriptome analysis of venom glands from a single fishing spider *Dolomedes mizhoanus*. Toxicon 73: 23-32.

Krupa JJ, Sih A (1998) Fishing spiders, green sunfish, and a stream-dwelling water strider: male-female conflict and prey responses to single versus multiple predator environments. Oecologia 117: 258-265.

Lapinski W, Tschapka M (2013) Habitat use in an assemblage of Central American wandering spiders. J Arachnol 41: 151-159.

Leroy J, Leroy A (2003) Spiders of Southern Africa. Cape Town: Struik Publishers. 96 p.

McKeown KC (1943) Vertebrates captured by Australian spiders. Proc R Zool Soc NSW 1942/43: 17-30.

Meehean OL (1934) Spiders that fish. Nat Hist 34: 538-540.

Mohrhardt D (1963) (Photograph of *Dolomedes scriptus* preying on minnow). Turtox News 41: 197.

Pickard-Cambridge FOP (1903) On some new species of spiders belonging to the families Pisauridae and Senoculidae; with characters of a new genus. P Zool Soc Lond 73: 151-168.

Platnick NI (2013) The world spider catalog, version 14.0. American Museum of Natural History. Available: <http://research.amnh.org/iz/spiders/catalog/>. Accessed 18 October 2013.

Romand R (1980) Die Schutzanpassung von *Aphyosemion walkeri* im Waldgebiet der Elfenbeinküste. D.A.T.Z. 33: 262-264.

Smith H (2000) The status and conservation of the fen raft spider (*Dolomedes plantarius*) at Redgrave and Lopham Fen National Nature Reserve, England. Biol Conserv 95: 153-164.

Suhr JM, Davis JD (1974) The spider *Dolomedes sexpunctatus* as a predator on mosquitofish, *Gambusia affinis*, in Mississippi. Ass Southeast Biol Bull 21: 87.

Zahl P (1971) What's so special about spiders? Natl Geogr 140: 190-219.
