# Supplementary material for: Fish Predation by Semi-Aquatic Spiders: A Global Pattern
Source: PLoS One. 2014 Jun 18;9(6):e99459. doi: 10.1371/journal.pone.0099459 (PMC4062410; doi:10.1371/journal.pone.0099459)
Supplement: Flow Diagram S1 — PRISMA 2009 Flow Diagram. (DOC) [file pone.0099459.s002.doc]

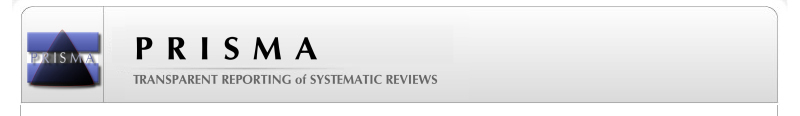
**PRISMA 2009 Flow Diagram**

**Screening**

**Included**

**Eligibility**

**Identification**

Records identified through database searching
(n = 74)

Additional records identified through other sources
(n = 45)

Records after duplicates removed
(n = 112)

Records screened
(n = 112)

Records excluded
(n = 9)

Excluded because reported incidences did not fulfill the criterion of ‘predation’ (n = 8) or because record was questionable (n = 1)

Records assessed for eligibility
(n = 103)

Full-text articles excluded, with reasons
(n = 14)

Reason for exclusions:

incidences witnessed under staged, unnatural conditions

Records included in the review
(n = 89)

Data set (n = 89, compiled in Table 1) used to generate Fig. 8

n = 82 records used to generate global map Fig. 1 (after exclusion of records with missing GPS coordinates)
