# Supplementary material for: Fish Predation by Semi-Aquatic Spiders: A Global Pattern
Source: PLoS One. 2014 Jun 18;9(6):e99459. doi: 10.1371/journal.pone.0099459 (PMC4062410; doi:10.1371/journal.pone.0099459)
Supplement: Checklist S1 — PRISMA 2009 Checklist. (DOC) [file pone.0099459.s003.doc]

| **Section/topic** | **#** | **Checklist item** | **Reported on page #** |
| --- | --- | --- | --- |
| **TITLE** | | |  |
| Title | 1 | Fish Predation by Semi-Aquatic Spiders: A Global Pattern  (this is a systematic review) | Title page |
| **ABSTRACT** | | |  |
| Structured summary | 2 | ***Background:*** Spiders, as a whole, are traditionally viewed as the classic example of a terrestrial predator that feeds on insects. Yet some semi-aquatic spiders are well-adapted for life near, or on, the water surface and the largest among them have been reported in the literature to occasionally catch and eat small fish. However, the fish catching capability of such spiders ist still largely unexplored. The article aims to assess the fish catching capability of such spiders on a global scale.  ***Search Strategy:*** Thomson-Reuters database (Web of Science), Google Web Search, Google Scholar, Google Books, Google Pictures, and ProQuest Dissertations & Theses were searched for reports on fish-catching spiders until June 2013, using key words such as ‘semi-aquatic spiders’, ‘fishing spiders’, ‘Dolomedes’, ‘Pisauridae’, ‘fish’, ‘piscivory’, ‘predation’, ‘nutritional ecology’, ‘food-web ecology’ etc. Furthermore, inquiries among biologists were undertaken for unpublished reports.  ***Results:***  A total of 89 records was included. We provide evidence that fish predation by semi-aquatic spiders is geographically widespread, occurring in many parts of the world though largely limited to the warmer regions between 40° S and 40° N. The captured fish usually range from 2-6 cm in length. Naturally occurring fish predation has been witnessed in more than a dozen spider species from the superfamily Lycosoidea, in two species of the superfamily Ctenoidea, and in one species of the superfamily Corinnoidea. Our finding that such a large diversity of spider taxa are engaged in fish predation is novel.  ***Conclusions:*** The capture and consumption of fish by spiders represents a significant departure from the average dietary patterns and predator-prey size ratios reported in the literature for spiders and fish might be an occasional prey item of substantial nutritional importance for semi-aquatic spiders. A better understanding of the nutritional ecology of the semi-aquatic spiders and their ecosystem role is needed. | Abstract |
| **INTRODUCTION** | | |  |
| Rationale | 3 | Photographic evidence supporting the existence of fish-catching by semi-aquatic spiders has been published in the past, but published accounts of open-field assessment of fish predation are often anecdotal, from very old literature sources and originate from only few locations. Additionally, the majority of published photographic sources depict spiders preying upon fish in captivity (staged situations). Thus, the propensity for spiders to feed on fish and the importance of this trophic relationship under natural conditions remains unclear. We conducted a global analysis of all available literature on fish predation by spiders to provide a broad, conceptual framework for this trophic relationship placed within the context of spider behavior and nutritional ecology. | Introduction |

| Objectives | | 4 | | The main objectives of the study were to determine;  - how common is fish predation by spiders under natural conditions?  - how geographically widespread are reported incidences of fish predation (i.e. globally)? and  - how widespread within spiders is this type of feeding behavior (i.e. is it confined to certain families)?. | | Introduction |
| --- | --- | --- | --- | --- | --- | --- |
| **METHODS** | | | | |  | |
| Protocol and registration | 5 | | We provide a ‘Detailed Reports Description’ (see SUPPORTING INFORMATION FILE S1). Apart from that, the senior author is in possession of all documents (e.g., data sheets, Email messages from taxonomists identifying fish or spider species depicted in photos, copies of peer-reviewed papers, internet reports, and other relevant documents) containing information included in the review. | | Methods section | |
| Eligibility criteria | 6 | | The records used in our investigation have been extracted from scientific papers and other reports originating from 1835 until 2013. About 98% of the records originate from papers/reports written in English and the reminder 2% from papers written in other languages which have been translated for us.  The 89 records included in our investigation had to fulfill the definition of ‘predation’ or ‘predation attempts’ (i.e., a fish being killed and devoured by a spider). For the most part, only incidences of fish predation by free-living spiders have been included; however, a few incidences where spiders killed fish in aquaria after wandering into buildings under non-staged conditions have been included to demonstrate these spiders’ high behavioral flexibility. Staged observations of captive spiders predating fish were not included in the core data set (Table 1) used to generate Fig. 1 (global distribution pattern) and Fig. 8 (relative importance of different spider families as fish predators). A few records of staged observations, however, were included in separate tables (Tables 2-3) and used in the Results and Discussion sections. | | Methods and Results sections | |
| Information sources | 7 | | Our extensive bibliographic search was based largely on the Thomson-Reuters database (Web of Science), Google Scholar, Google Books, and ProQuest Dissertations & Theses. In addition, an internet search for information on this topic was conducted; authors of reports on fish predation by spiders were contacted to obtain additional information on these incidences. Furthermore, inquiries among biologists were undertaken for unpublished reports on this topic. This way 89 records of fish predation by spiders were gathered (Table 1), 44 of which (49%) were previously reported in the scientific literature.  Unpublished photographs of fish predation events made available to us by biologists / photographers were sent to established ichthyologists and spider taxonomists for identification of fish and spiders, respectively (see Acknowledgements section for a list of scientists engaged in these activities). Unless reported in the literature or by the respondents in our survey, the total lengths of fish prey were estimated based on photographs (the approximate spider size known from the literature serving as a standard [replacing a reference scale]; see Table 1). Report numbers used in the tables refer to the respective detailed report description (SUPPORTING INFORMATION FILE S1). | | Methods and Acknowledgements sections | |
| Search | 8 | | Our search was based on large databases such as for instance the Thompson-Reuters database using key words such as ‘semi-aquatic spiders’, ‘fishing spiders’, ‘Dolomedes’, ‘Pisauridae’, ‘fish’, ‘piscivory’, ‘predation’, ‘nutritional ecology’, ‘food-web ecology’ etc.. | | Methods section | |
| Study selection | 9 | | This is explained under item 10. | | Methods section | |
| Data collection process | 10 | | A total of 119 records were collected through database searching. In 74 cases the records originated from the scientific literature and in 45 cases the records were identified through other sources (documents posted on the internet, personal communications by fish biologists, arachnologists, ecologists, photographers etc.).  In several cases, the authors of scientific studies and internet documents were contacted to obtain additional information.  The 119 records were carefully studied and compiled in a Table which was later used to identify and remove any dublicates. In this way n = 7 dublicates were identified and removed. Thereafter a total of n = 112 records were left for further screening.  During the screening process n = 9 more records were deleted (mostly because records were questionable; for instance, captive spiders being fed dead fish which does not fulfill the criterion of ‘predation’). This resulted in n = 103 records to be assessed for eligibility.  Subsequently, n = 14 full-text articles had to be excluded with reasons. In these cases, fish predation was witnessed under unnatural conditions (staged observations of captive spiders).  Finally, a total of n = 89 records (compiled in Table 1) were included in the Systematic Review. Also see SUPPORTING INFORMATION FILE S1. | | Methods section | |
| Data items | 11 | | The following data have been extracted (as far as available) for each record:  - Predator (spider taxon)  - Prey (fish taxon)  - Estimated fish length / spider length ratio  - Type of evidence (‘Photo’ or ‘Direct Observation’ or ‘Unknown’)  - Country / Location (latitude / longitude) where an incidence has been witnessed  - Source (referring to the ‘References’ or to a particular ‘website’ or being indicated as a ‘personal communication from a particular scientist’)  Each record has been given a report number (referring to the SUPPORTING INFORMATION FILE S1, where a detailed description is provided)  → These data have been compiled in Table 1 and were used in the Results and Discussion section. | | Methods, Results and Discussion sections. | |
|  | |  | |
| Risk of bias in individual studies | 12 | | In cases where fish species and spider species had to be identified by taxonomists based on photos, the accuracy of the identifications depended on the quality and resolution of the photo material which varied from study to study. Hence, in some studies fish and spiders could be identified to species level with absolute certainty, but not in others and thus potentially creating some bias. To resolve this problem, we used “careful wording” when mentioning fish species and spider species in the text (such as for instance “.....adult *Ancylometes* sp. (probably *Ancylometes rufus*)….”). Thus, we feel that our statements are pretty safe. | | Methods section | |
| Summary measures | 13 | | We did not deal with this. | | - | |
| Synthesis of results | 14 | | See under item 21. | | - | |

| **Section/topic** | **#** | **Checklist item** | **Reported on page #** |
| --- | --- | --- | --- |
| Risk of bias across studies | 15 | The majority of incidences of spider predation upon fishes were reported from North America, especially the eastern part of USA, whereas fewer were reported from Africa, Asia, Australia, Europe or the Neotropics. To a large extent, the pattern shown in our study may simply reflect the distribution of potential observers and especially those with the capacity or propensity to report observations of spider predation. Thus, this disparity may potentially bias our view of geographical patterns of the incidence of fish predation by spiders. We have very carefully qualified our statements about geographic distribution of predation to ensure that the reader is aware of this potential bias. | See Discussion section |
| Additional analyses | 16 | No additional analyses have been conducted. | - |
| **RESULTS** | | |  |
| Study selection | 17 | In total 89 records of fish predation by spiders have been included which were obtained from 67 papers / reports (Table 1; SUPPORTING INFORMATION FILE S1). | Results section |
| Study characteristics | 18 | The variables extracted from each paper / report (item 11) are presented in Table 1 (including citations). | Results section |
| Risk of bias within studies | 19 | See item 12. | Methods section |
| Results of individual studies | 20 | The main results of the individual studies (see SUPPORTING INFORMATION FILE S1) are comparatively presented in Table 1. | Results section |
| Synthesis of results | 21 | ***Geographic Distribution of Fish Predation by Spiders:*** Fish capture by spiders has been reported from all continents with the exception of Antarctica (~90% of the incidences occurring in the warmer areas of the globe between latitude 40° S and 40° N)(Fig. 1) and were typically observed at the margin of freshwater streams, rivers, creeks, bayous, lakes, ponds, swamps, and fens.  Fish predation by spiders has been most frequently documented in North America (52% of the total). Multiple incidences of fish predation have been reported from the Neotropics (14 reports) and Australia (12 reports). Less common are reports from Asia (7 reports), Africa (6 reports), and Europe (4 reports).  ***Spiders Engaged in Fish Predation:*** The superfamilies Lycosoidea and Ctenoidea are those documented as preying upon fish under open-field conditions (~80% of the observed predation events being attributable to Pisauridae) (Figs. 2-8; Table 2).  ***Fishes Captured by Spiders:*** All 89 cases of fish predation listed in Table 1 involve small freshwater fish belonging predominantly to the 2-6 cm size class (also see Table 3).  ***Predator–Prey Size Ratio:*** Results herein show that semi-aquatic spiders captured fish prey whose body length usually exceeded the spiders’ body length (the captured fish being, on average, 2.2 times as long as the spiders). This contrasts markedly with predator/prey ratios reported for spiders feeding on insects. | Results section |
| Risk of bias across studies | 22 | See Item 15. | Discussion section |
| Additional analysis | 23 | No such additional analyses have been conducted. | - |
| **DISCUSSION** | | |  |
| Summary of evidence | 24 | In this paper a phenomenon is described which is novel (namely that fish predation by spiders is much more widespread than previously thought) and a comprehensive explanation (at least in terms of hypotheses) is provided. We were looking at this novel phenomenon from different angles and accordingly the Discussion section is divided into subsections by subtitles.  In a first subsection we deal with "scavenging vs. predation". In the older literature it has often been argued that fish eating events in semi-aquatic spiders might be simply cases of scavenging (i.e., spiders coming across dead fish floating on the water surface). Thus, we felt that it is important to define the term ‘predation’ (which includes the killing and devouring of a prey animal) and to explain that it takes powerful neurotoxins / enzymes enabling the spiders to kill / digest fish that often exceed them in size.  In a next subsection we discuss the global distribution pattern of fish predation by spiders. We explore the potential for observer bias (specifically the number of observers and propensity to report) and conclude that it may be important especially with respect to the reporting frequencing of prediation in the Neotropcs and in Asia. We address such questions as: why are there more such incidences in the south than in the north, why are there more such incidences in eastern compared to western USA, and why are reports from Europe so rare? The high diversity of species within families reported elsewhere to be engaged in fishing (e.g. within Asia) suggests that although fish predation is widespread it is likely to be underreported.  In a further subsection we discuss the nutritional importance of fish predation by spiders (see Table 4) and in a last subsection we deal with this topic from a point of view of food-web ecology. | Results and Discussion sections |
| Limitations | 25 | The limitations of this review are that the feeding habits of large semi-aquatic spiders have been underreported in some regions of the globe (e.g., in the Neotropics large semi-aquatic spiders occur most commonly in remote areas of tropical rainforest and are strictly nocturnal as adults, characteristics that make their observation in the wild very difficult).  A further limitation may be that scientific papers / reports published in languages other than English (e.g. in Chinese and Russian) have largely been ignored by us due to the language barrier resulting in a lack of information referring to certain parts of the globe.  Further, our analysis allowed us to raise several hypotheses about fish predation and its importance but these remain untested. We expect that this review will sharpen the focus on this group of spiders and encourage rigorous research on this phenomena. | Discussion section |
| Conclusions | 26 | The capture and consumption of fish by spiders represents a significant departure from the average dietary patterns and predator-prey size ratios reported in the literature. This phenomena is geographically widespread and occurs across a range of spider families of larger body mass. Fish might be an occasional prey item of substantial nutritional importance for semi-aquatic spiders especially with regard to reproductive requirements. A better understanding of the nutritional ecology of the semi-aquatic spiders and their ecosystem role is needed. This study is a pioneer study leaving open many questions, but we hope to stir up the discussion and generate strong interest in the feeding biology of semi-aquatic spiders leading to new research projects in the near future. | Discussion and Conclusions sections |

| **FUNDING** | | |  |
| --- | --- | --- | --- |
| Funding | 27 | No current funding sources for this study. | - |

*From:*  Moher D, Liberati A, Tetzlaff J, Altman DG, The PRISMA Group (2009). Preferred Reporting Items for Systematic Reviews and Meta-Analyses: The PRISMA Statement. PLoS Med 6(6): e1000097. doi:10.1371/journal.pmed1000097

For more information, visit: **www.prisma-statement.org**.

Page 2 of 2
